# A bibliometric study on the impact of gut microbiota on the efficacy of immune checkpoint inhibitors in cancer patients: analysis of the top 100 cited articles

**DOI:** 10.3389/fimmu.2024.1519498

**Published:** 2025-01-16

**Authors:** Ziqi Zhao, Kun Xu, Boqian Hu, Yizhuo Jiang, Xisheng Xu, Yuliang Liu

**Affiliations:** ^1^ School of Basic Medicine, Zhejiang Chinese Medical University, Hangzhou, China; ^2^ Hebei Provincial Hospital of Traditional Chinese Medicine, Hebei University of Chinese Medicine, Shijiazhuang, China

**Keywords:** bibliometrics analysis, cancer patients, gut microbiota, immune checkpoint inhibitors, immunotherapy

## Abstract

**Background:**

Immune checkpoint inhibitors (ICIs) have transformed oncological treatment by modulating immune responses against tumors. However, their efficacy is subject to inter-patient variability and is associated with immune-related adverse events (irAEs). The human gut microbiota, a complex microbial ecosystem, is increasingly implicated in modulating responses to ICIs. This bibliometric analysis examines the 100 most-cited articles to elucidate trends and advancements in research concerning the gut microbiota’s impact on ICI efficacy.

**Methods:**

A systematic literature retrieval was conducted within the Web of Science Core Collection (WoSCC), focusing on the 100 most-cited articles. VOSviewer and CiteSpace were utilized for bibliometric analysis, examining collaborative patterns and keyword co-occurrences. The relationship between citing and cited entities was analyzed, and burst ranking identified research hotspots based on citation frequency.

**Results:**

The 100 most-cited publications encompassed a range of disciplines, with a predominance of oncological research. The United States and China were leading in publication volume, with France and Canada also contributing significantly. French institutions, particularly INSERM and Université Paris Cite, were prolific. Routy, Bertrand and Zitvogel, Laurence were prominent among high-impact authors. Dominant keywords included “gut microbiota,” “immunotherapy,” “efficacy,” and “cancer.” The article by Routy et al. (2018) was the most frequently cited.

**Conclusions:**

This study highlights the significant role of the gut microbiota in ICI development and efficacy, emphasizing the necessity for international and interdisciplinary collaboration. The research is progressively focusing on managing immunotherapy side effects and optimizing treatment strategies. Challenges, including individual variability in gut microbiota composition, persist. Further research is imperative to exploit the potential of the gut microbiota in cancer therapy, advocating for personalized approaches and a more profound comprehension of the underlying mechanisms.

## 1 Introduction

Immune checkpoint inhibitors (ICIs) represent a revolutionary cancer treatment modality that plays a crucial role in the fight against cancer. They work by lifting the brakes that cancer cells use to suppress the immune system, thereby enhancing the body’s immune response against tumors. Immune checkpoints are a natural regulatory mechanism within the immune system, designed to prevent overactivation of immune cells and protect healthy tissues from damage (1). However, cancer cells sometimes exploit these checkpoint pathways to evade surveillance and attack by the immune system. The main immune checkpoints currently known include Cytotoxic T-Lymphocyte Antigen 4 (CTLA-4) (2), Programmed Death Protein 1 (PD-1) (3), and its ligand (PD-L1) (4). The primary mechanism of action of ICIs involves blocking these inhibitory signals, which enhances the activity of T cells, promotes the formation of memory T cells, and subsequently boosts the immune response against cancer cells. The discovery and clinical application of ICIs have marked a significant leap in the field of cancer treatment. However, they also present certain challenges. Not all cancer patients respond to ICIs, and these drugs can potentially cause immune-related adverse events (irAEs) (5). Additionally, some tumors may gradually develop resistance to ICIs. These challenges are currently present and being addressed by ongoing research and clinical efforts.

The gut microbiota refers to the community of microorganisms that reside in the human intestine, co-evolving with the host to form a complex ecosystem. These microbes are not only involved in the digestion and absorption of food but also help maintain the body’s homeostasis through interactions with the immune system (6). The composition of the gut microbiota can serve as a potential biomarker for predicting the response of cancer patients to immune checkpoint inhibitor therapy. Specific types of gut bacteria, such as certain species of *Bifidobacterium*, have been shown to be associated with more effective responses to immunotherapy (7). Metabolic products produced by the gut microbiota, such as short-chain fatty acids, can affect the tumor microenvironment and potentially modulate the activity of immune cells (8, 9). These metabolites may help enhance the ability of immune cells to attack tumors, thereby improving the efficacy of immunotherapy. In addition, the gut microbiota can also influence the development and migration of immune cells, especially regulatory T cells (Tregs) and other immunosuppressive cells (10). The accumulation of these cells in the tumor microenvironment may suppress the immune response and thus affect the outcome of immunotherapy. Therefore, the gut microbiota may impact the efficacy of ICIs through various mechanisms, which deserves further exploration and consideration in cancer treatment strategies.

Although existing literature has emphasized the link between the gut microbiota and ICIs, to our knowledge, no studies have systematically employed bibliometric methods to analyze the development trends of the most representative publications in this field. Therefore, the purpose of this study is to conduct a comprehensive analysis of the top 100 most-cited publications on the research of gut microbiota affecting the efficacy of ICIs in cancer patients using bibliometric methods. Our aim is to sort out the current state of research and predict future development trends, providing valuable references and in-depth insights for researchers in this field.

## 2 Materials and methods

### 2.1 Data collection and sources

Bibliometrics is a powerful methodological tool that can reveal the development trends of a specific discipline or research field over a certain period of time. To ensure the accuracy and authority of the research results, choosing a comprehensive and representative database is crucial. The Web of Science (WoS), as a multidisciplinary comprehensive database, includes many high-impact scientific journals and world-class indexes. Compared to Scopus or MEDLINE/PubMed, WoS can provide more comprehensive information for bibliometric analysis (11, 12).

Among the many databases, the Web of Science Core Collection (WoSCC) is highly regarded for its comprehensiveness, systematicity, and authority. It covers numerous prestigious academic journals, making the publications in the WoSCC database largely reflective of current research trends. In a wide range of research practices, the WoSCC database has been proven to be the preferred resource for bibliometric research (13).

However, it is worth noting that the WoSCC integrates multiple sub-databases, and not all of these sub-databases are suitable for bibliometric analysis. Based on past research experience, the Science Citation Index Expanded (SCI-E) is considered the preferred database for bibliometric analysis due to its broad acceptance, wide application range, and high applicability (14).

Based on the considerations mentioned above, we have decided to use the Science Citation Index Expanded (SCI-E) within the Web of Science Core Collection (WoSCC) as the data source for our research. This choice is aimed at ensuring that we can obtain the most accurate and representative data, thereby delving deeper and deriving valuable research insights.

### 2.2 Search strategy and criteria

To ensure the accuracy of our research and to mitigate potential biases from database updates, we coordinated two researchers to independently search for papers on the application of gut microbiota in the field of immune checkpoint inhibitor research. The search and data collection were efficiently completed within a single day. The data collected included the titles, keywords, abstracts, authors, institutions, and references of the articles, all of which were obtained and saved in plain text format. The specific search strategy is as follows:

(TI=(“immune checkpoint inhibitors” OR “immune checkpoint blockade” OR “immunological checkpoint inhibitor” OR “immune checkpoint inhibitor” OR “immunological checkpoint inhibitors” OR “immuno-checkpoint inhibitors” OR “immune checkpoint blockers” OR”AntiCTLA-4” OR “Anti-PD-1” OR “Anti-PD-L1” OR “Ipilimumab” OR “Tremelimumab” OR “Pembrolizumab” OR “Atezolizumab” OR ICIs) OR AB=(“immune checkpoint inhibitors” OR “immune checkpoint blockade” OR “immunological checkpoint inhibitor” OR “immune checkpoint inhibitor” OR “immunological checkpoint inhibitors” OR “immuno-checkpoint inhibitors” OR “immune checkpoint blockers” OR”AntiCTLA-4” OR “Anti-PD-1” OR “Anti-PD-L1” OR “Ipilimumab” OR “Tremelimumab” OR “Pembrolizumab” OR “Atezolizumab” OR ICIs)) AND (TI = ((intestinal OR gut) NEAR/1 (microflora* OR microbiota* OR flora OR microbiome)) OR AB=((intestinal OR gut) NEAR/1 (microflora* OR microbiota* OR flora OR microbiome)))

**Figure 1:**
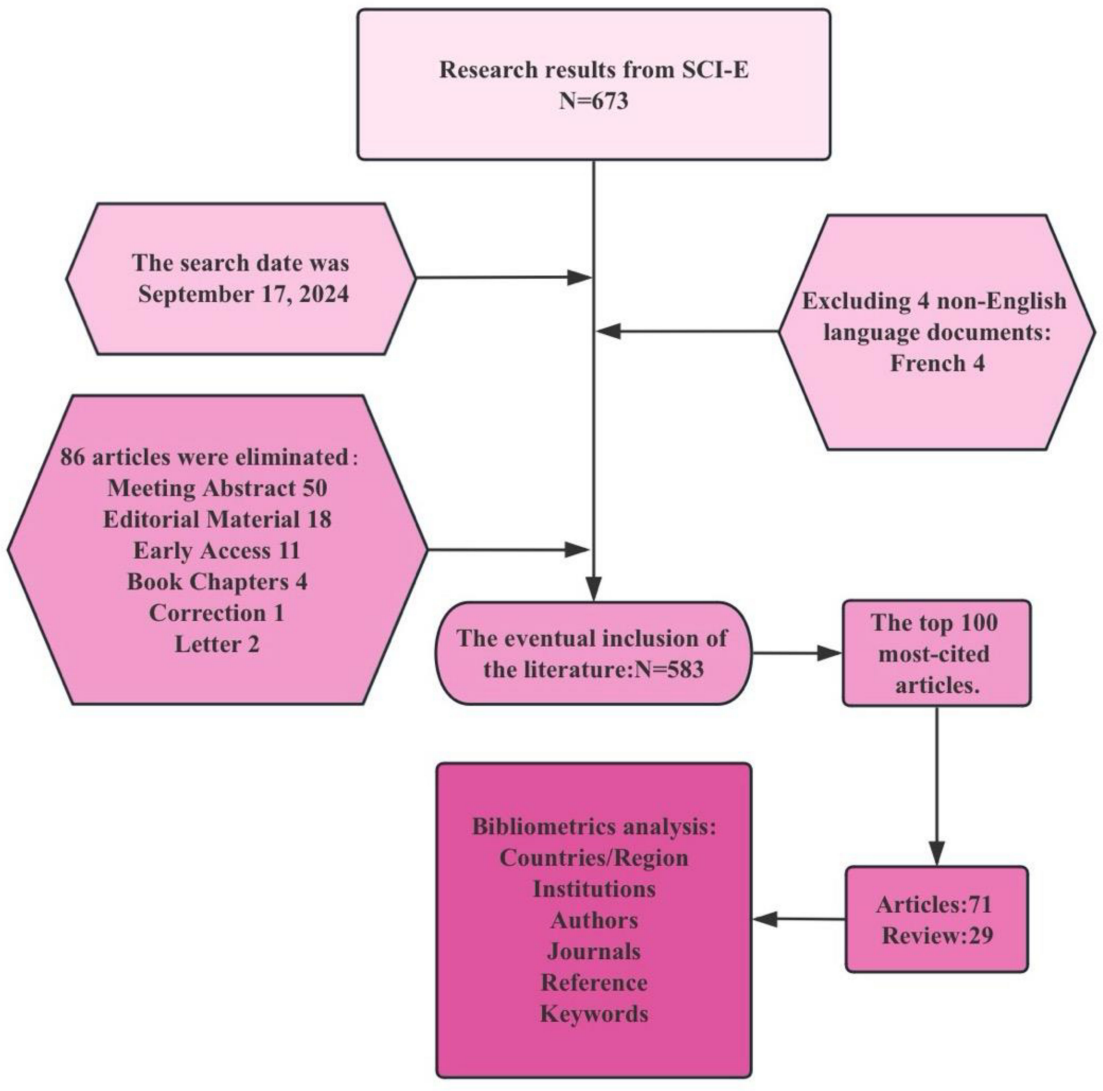
A flow chart of the retrieval process in this study.

The search was conducted on September 17, 2024, with detailed steps as shown in **Figure 1**.

### 2.3 Manual screening process

Based on the specific requirements of this study, we designed a set of clear inclusion criteria: papers eligible for analysis must meet the following conditions (1): the language must be English (2); the article types are limited to original research and review articles (3); the content of the study focuses on the field of ICIs, involving the application of gut microbiota. Through the initial search strategy, we preliminarily screened 673 relevant papers. Subsequently, based on the aforementioned criteria, we conducted a more detailed manual screening. To ensure the accuracy and effectiveness of the screening results, this step was carried out independently by two researchers, who organized and discussed any uncertain literature encountered during the screening process to determine whether it should be included in the final scope of the study. We then ranked these publications by citation frequency from highest to lowest. In cases where citation frequencies were the same, we prioritized publications with more recent publication dates to ensure that the study reflects the latest academic trends. Ultimately, we selected the top 100 articles with the highest citation frequency for in-depth analysis, which included 71 original research articles and 29 review articles. The top 100 publications by citation frequency included in the final analysis are listed in **Supplementary Table S1**.

### 2.4 Bibliometric analysis methodology

In this study, we primarily utilized three tools for bibliometric analysis and visualization: VOSviewer, CiteSpace, and the bibliometrix-R package. VOSviewer, a widely used bibliometric tool, is known for its core function of revealing the structure and development of knowledge domains by constructing and visualizing the network relationships between literature (15). In this research, we employed VOSviewer to conduct a co-authorship network analysis for entities such as countries, institutions, and authors, aiming to understand and display the interconnections between these projects.

CiteSpace, another mainstream analysis and visualization software in the field of bibliometrics (16), was used in this paper to delve into the role and impact of gut microbiota in the research field of ICIs. Its core functionality involves constructing centrality graphs, journal co-occurrence overlays, and analyzing keyword clustering, timelines, etc., to identify hot topics and frontier issues in the field. Through CiteSpace, we have predicted potential future development trends, providing new perspectives and directions for research in this area.

In addition, to analyze trends in collaborative between countries, we used the bibliometrix-R package of bibliometric analysis tools (17). Furthermore, we used Microsoft Excel to perform frequency statistics on the selected analysis items and constructed tables based on these statistics.

### 2.5 Related parameter settings

Based on the analysis project, the node types were set to “countries, authors, co-cited authors, or keywords”; the time slice was set to 1 year; the k value was set to 25; in the Pruning section, we selected “Pathfinder,” “Pruning sliced networks,” and “Pruning the merged network”; other settings were kept at default. In the charts, the width of the lines in different shades is proportional to the publication volume, and the color of the nodes represents different periods. “Cool” shades represent earlier publication dates, while “warmer” colors represent later publication dates, and the color of the lines connecting the nodes follows the same rule.

In co-occurrence and collaboration maps, “centrality” refers to the betweenness centrality of the nodes; if the centrality value is greater than 0.1, the node can be considered a key node; “significance value” is used to measure the importance of the nodes; in clustering maps, the smaller the serial number of the cluster, the larger the scale of the cluster; “size” refers to the number of nodes included in the cluster; the “silhouette coefficient” (S) is a measure of the average silhouette value of the cluster, and when the S value is greater than 0.7, the clustering effect is considered good, indicating high similarity among all nodes and highly persuasive results; the “modularity” (Q) of a cluster module is reflected in its numerical value, and a Q value greater than 0.3 indicates that the clustering structure is significant; in the bubble chart, the radius of the bubbles represents the total number of citations, the values on the X-axis represent the number of publications, the values on the Y-axis represent the average citation count, and different colors represent different years.

## 3 Results

### 3.1 Data fundamentals

Through a meticulously designed search strategy and rigorous screening process, we have selected the top 100 most-cited papers on the application of gut microbiota in the field of ICIs from the Web of Science Core Collection (WoSCC) database. We conducted a comprehensive statistical analysis of the field for these papers and provided a detailed summary of their research findings.

Based on the statistics and analysis of the original literature data conducted through WoSCC, we have compiled **Table 1**. This table lists in detail the proportion of papers published in each research field, arranged in descending order. It is evident that Oncology, which accounts for 42%, is the primary category in this area of research. This indicates a significant interest in the role of gut microbiota in cancer therapy, particularly in the application of ICIs. The presence of Experimental Medicine Research and Biochemistry & Molecular Biology in the top ten, with 14% and 13% respectively, highlights the in-depth investigation into the interaction between gut microbiota and ICIs in the realms of basic science and experimental research. Multidisciplinary Sciences also constitutes 13%, suggesting that research in this field encompasses various disciplines, potentially including microbiology, genetics, pharmacology, etc., underscoring the significance of interdisciplinary collaboration. These data in the table indicate that the application of gut microbiota in the field of ICIs is a multidisciplinary and cross-domain research hotspot, touching on multiple areas such as oncology, immunology, basic biology, and digestive system diseases. The diversity and depth of research demonstrate that scientists are actively exploring how gut microbiota can influence the efficacy of ICIs and how to leverage this knowledge to enhance cancer treatment.

**Table 1:** Ranking of web of science categories for the top 100 cited articles.

Rank	Web of Science Categories	Record Count	% of 100
1	Oncology	42	42%
2	Immunology	20	20%
3	Medicine Research Experimental	14	14%
4	Biochemistry Molecular Biology	13	13%
5	Multidisciplinary Sciences	13	13%
6	Cell Biology	10	10%
7	Gastroenterology Hepatology	10	10%
8	Microbiology	4	4%
9	Chemistry Multidisciplinary	3	3%
10	Urology Nephrology	3	3%
11	Hemaology	2	2%
12	Medicine General Internal	2	2%
13	Parasitology	2	2%
14	Respiratory System	2	2%
15	Virology	2	2%
16	Biophysics	1	1%
17	Chemistry Physical	1	1%
18	Engineering Biomedical	1	1%
19	Genetics Heredity	1	1%
20	Materials Science Multidisciplinary	1	1%
21	Nanoscience Nanotechnology	1	1%
22	Pharmacology Pharmacy	1	1%
23	Physics Applied	1	1%
24	Physics Condensed Matter	1	1%

### 3.2 Overview of publishing year and citations

**Figure 2:**
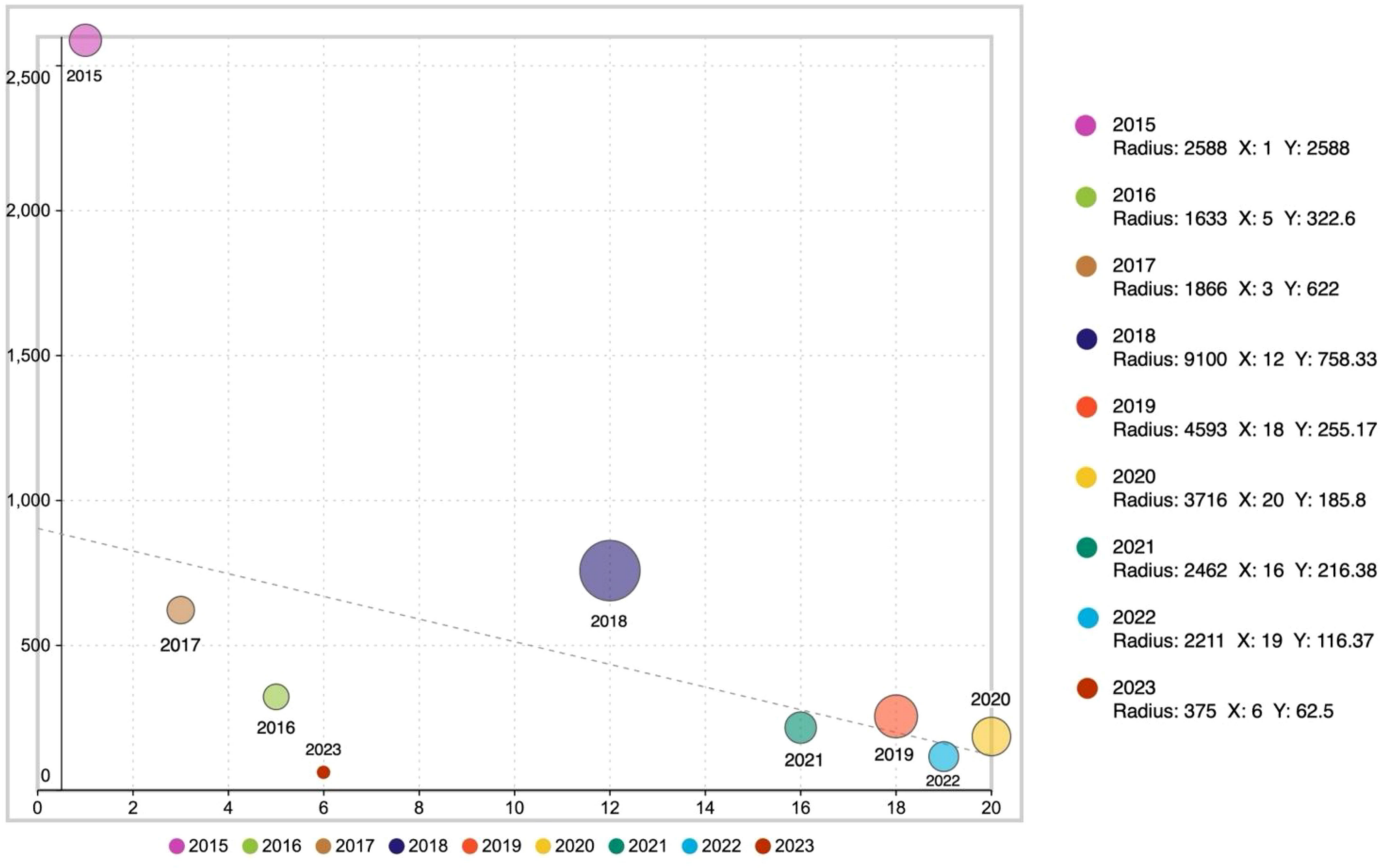
Bubble chart of top 100 gut microbiota articles in ICIs field by citation frequency.

Among the 100 publications included, the publication dates span from 2015 to 2023. To visually present this data, we constructed a chart where the x-axis represents the number of publications, the y-axis represents the average number of citations, and the total number of citations is indicated by the size of the bubbles. Additionally, we used different colors to distinguish between different publication years, making the relationships between the data clear at a glance in **Figure 2**. In the data labels, we detailed the total number of citations as well as the specific values for the x-axis and y-axis.

By analyzing **Figure 2**, we can observe that the total number of citations in 2018 reached 9,100 times, the highest among all years. The year 2020 leads with 20 publications. Meanwhile, the highest average number of citations was in 2015, with 2,588 times. In contrast, the year 2023 has the lowest values, with 6 publications, an average of 62.5 citations per publication, and a total of 375 citations. These data provide us with an in-depth insight into the development trends of the research field.

### 3.3 Countries/region analysis of production

**Figure 3:**
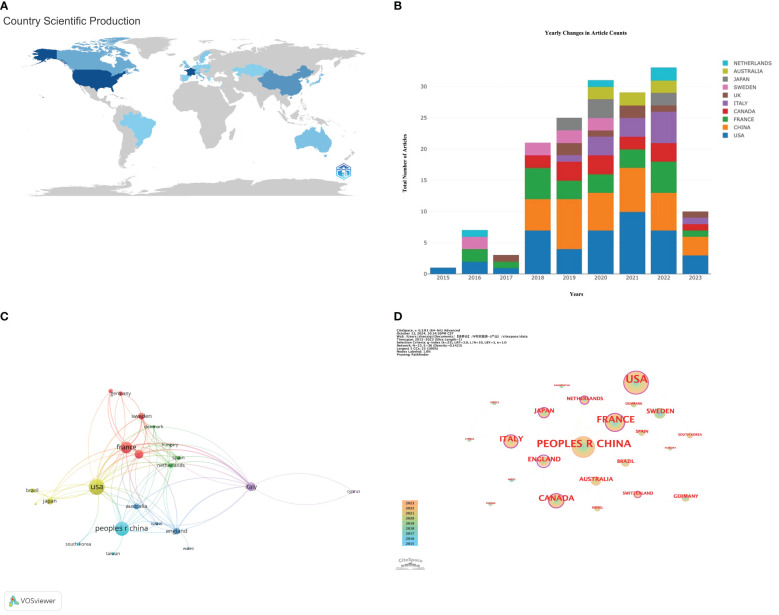
**(A)** World map based on the total publications of different countries/regions; **(B)** Yearly publication volume trend of the top 10 countries/regions from 2015 to 2023. **(C)** Citation network visualization map of countries/regions; **(D)** Map of the centrality of national cooperation.

Researchers from 23 countries around the globe have collectively contributed a wealth of scholarly outcomes in the interdisciplinary study of gut microbiota applications in the field of ICIs. The geographical distribution of these achievements is clearly depicted in the map shown in **Figure 3A**. **Table 2** is sorted by the number of published articles from high to low. Among the 100 most-cited articles, there are 5 countries with more than 10 publications, namely: the United States (42), China (35), France (23), Canada (14), and Italy (13).

**Table 2:** Global publication counts and centrality rankings of countries.

Sort by the Number of Publications						Sort by Centrality		
Rank	Country	Count	Citations	TLS	Centrality	Country	Centrality	Count
1	USA	42	20303	58	0.2	United Kongdom	1.24	8
2	China	35	5907	22	0	Nertherlands	0.37	4
3	France	23	11501	52	0.33	Canada	0.36	14
4	Canada	14	2970	41	0.36	Japan	0.35	7
5	Italy	13	2250	35	0.33	France	0.33	23
6	United Kongdom	8	1993	18	1.24	Italy	0.33	13
7	Sweden	8	6364	24	0.09	USA	0.2	42
8	Japan	7	1865	9	0.35	Switzerland	0.17	3
9	Australia	6	910	14	0.05	Sweden	0.09	8
10	Netherlands	4	543	16	0.37	Australia	0.05	6
11	Germany	4	1543	7	0	Spain	0.05	3
12	Brazil	4	3349	6	0	Kazakhstan	0.05	1
13	Switzerland	3	273	6	0.17	Israel	0.03	2
14	Spain	3	457	14	0.05	Hungary	0.03	1
15	Israel	2	1213	7	0.03	China	0	35
16	South Korea	2	197	3	0	Germany	0	4
17	Denmark	2	1442	12	0	Brazil	0	4
18	Kazakhstan	1	705	2	0.05	South Korea	0	2
19	Hungary	1	232	9	0.03	Denmark	0	2
20	Wales	1	600	1	0	Wales	0	1
21	Poland	1	55	0	2	Poland	0	1
22	Greece	1	138	2	0	Greece	0	1
23	Cyprus	1	138	2	0	Cyprus	0	1

TLS, total link strength.

**Figure 3B** further reveals the differences in annual publication trends among the top ten countries by publication volume. It can be observed that the United States leads in terms of the number of articles, total citations, and total connectivity strength, demonstrating its significant influence in the field. Additionally, the United States is the only country that has consistently published research articles related to this field from 2015 to 2023, further highlighting its sustained leadership and research vitality in the area.

Using the VOSviewer tool, we were able to conduct an in-depth analysis of the research collaboration network between countries, as shown in **Figure 3C**. The thickness of the lines in the figure visually represents the closeness of the co-authorship relationships between countries; the thicker the line, the more frequent the collaborative research between the two countries. Based on the ranking of Total Link Strength (TLS), the United States, France, Canada, Italy, and Sweden are in the top five, demonstrating that these countries have particularly close cooperation in the field of research applying gut microbiota to ICIs.

By employing the CiteSpace software, we further revealed the central positions of various countries within the collaboration network, as depicted in **Figure 3D**. Nodes marked with purple circles, with centrality values exceeding 0.1, indicate that these countries play a more pivotal role within the network. Although the United Kingdom has a high centrality value of 1.24, demonstrating its very active role in collaborative research, its publication count ranks only 7th. At the same time, despite China ranking second in publication volume, its lack of centrality suggests that there is significant room for improvement in terms of international collaboration. **Table 2** provides further explanation and analysis.

### 3.4 Institutions analysis of production

**Figure 4:**
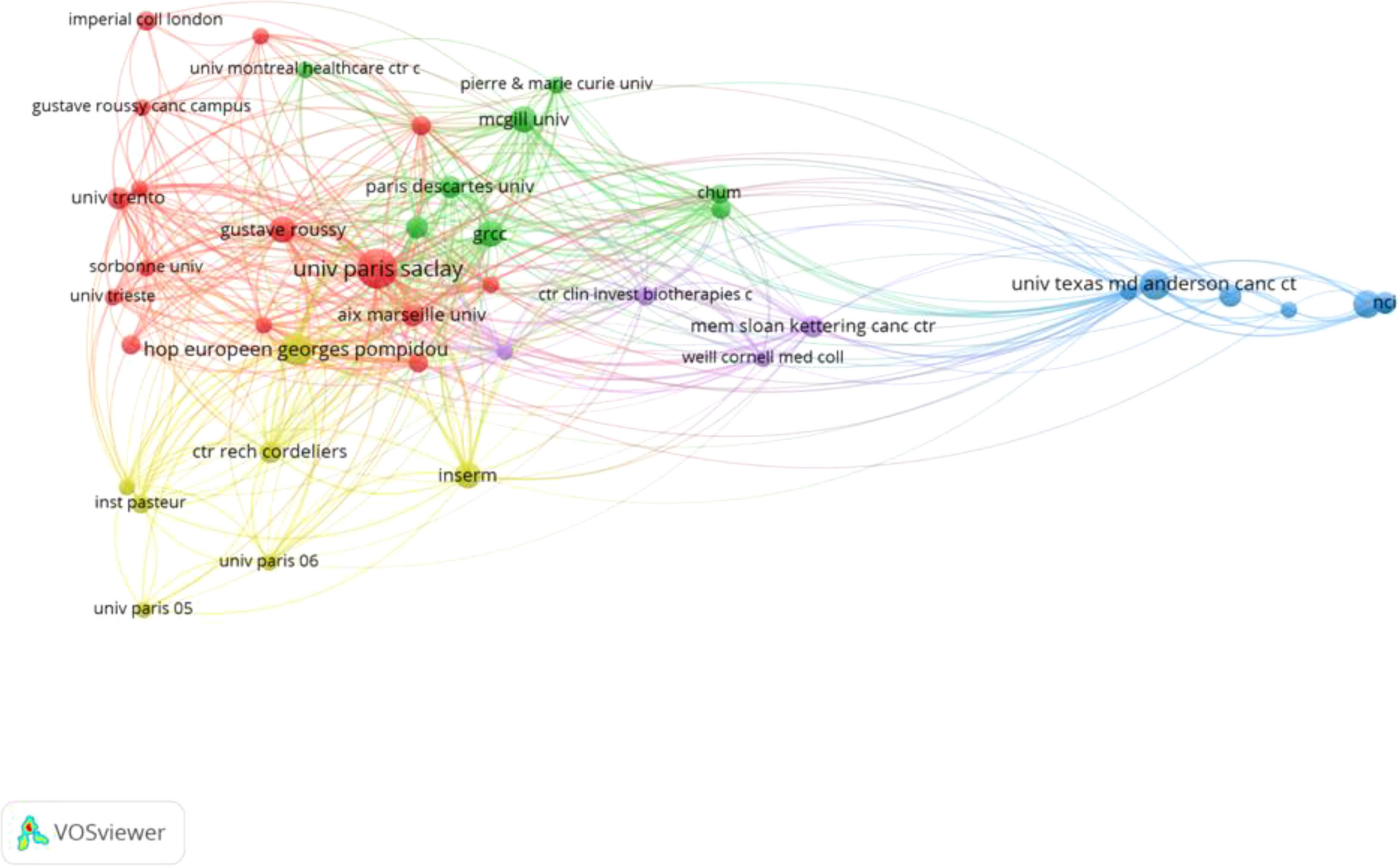
The visualization map of institutions co-authorship analysis generated by VOSviewer.

**Figure 4** and **Table 3** together demonstrate the most active institutions in the field of ICIs, specifically in the research on gut microbiota, among the top 100 most-cited studies. A total of 366 institutions and universities worldwide have conducted independent or collaborative research in this area. **Table 3** specifically lists 11 institutions with 9 or more publications, with 8 located in France, 2 in the United States, and 1 in Canada.

**Table 3:** Ranking of the top 11 institutions by number of publications.

Rank	Institution	Count	Centrality	Citations	Average Citation	Country
1	INSTITUT NATIONAL DE LA SANTE ET DE LA RECHERCHE MEDICALE INSERM	18	0.13	10905	605.83	France
2	UNIVERSITE PARIS CITE	18	0.06	8108	450.44	France
3	UNICANCER	17	0.1	10686	628.59	France
4	UNIVERSITE PARIS SACLAY	17	0.09	10839	637.59	France
5	GUSTAVE ROUSSY	16	0.03	10595	662.19	France
6	ASSISTANCE PTBLIQUE HOPITAUX PARIS APHP	13	0.02	7387	568.23	France
7	UNIVERSITY OF TEXAS SYSTEM	13	0	7322	563.23	USA
8	HOPITAL UNIVERSITAIRE EUROPEEN GEORGES POMPIDOU APHP	10	0.01	5778	577.80	France
9	SORBONNE UNIVERSITE	10	0	5688	568.80	France
10	UTMD ANDERSON CANCER CENTER	10	0	6686	668.60	USA
11	UNIVERSITE DE MONTREAL	9	0	2040	226.67	Canada

**Figure 4** presents a collaboration network consisting of 41 institutions that have established connections with other institutions having three or more publications. This network is composed of 41 nodes, 413 links, and 5 clusters. Our analysis reveals that these studies were primarily conducted through collaboration between researchers from universities or research institutions in France, the United States, and Canada. Among these institutions, the French National Institute of Health and Medical Research (Institut National de la Santé et de la Recherche Médicale, INSERM) and Paris University (UNIVERSITE PARIS CITE) have the highest number of publications in this field, with 18 each. Following closely are the University of Paris-Saclay (UNIVERSITE PARIS SACLAY) and the French Cancer Institute (UNICANCER), each with 17 publications, and both are also French institutions. These institutions have played a more significant role in the publication of these studies compared to others. Furthermore, the French National Institute of Health and Medical Research has the highest number of citations, reaching 10,905, followed by the University of Paris-Saclay with 10,839 citations. These figures not only highlight the research impact of these institutions in the field but also reflect their crucial role in global scientific collaboration. The research outcomes from these institutions have not only advanced science but also provided valuable references and insights for future research.

### 3.5 Authors and co-authors analysis

In the in-depth review of this study, we identified 1320 authors who directly contributed to the articles, as well as 4614 scholars who were widely co-cited in the literature. **Table 4** particularly highlights the top nine authors by publication count (with more than 5 publications) and the top ten co-cited authors by citation frequency. Routy, Bertrand and Zitvogel, Laurence lead with 14 publications each, followed by Derosa, Lisa and Kroemer, Guido, with 11 and 10 publications respectively.

**Table 4:** The 9 most productive authors and the top 10 co-cited authors with the highest citations.

Rank	Author	Country	Documents	Citations	TLS	Co-cited author	Centrality	Citations	Country
1	Routy, Bertrand	Canada	14	5997	99	Routy, Bertrand	0.04	76	Canada
2	Zitvogel, Laurence	France	14	5987	94	Gopalakrishnan, Vancheswaran	0	76	USA
3	Derosa, Lisa	France	11	4879	86	Vétizou, Marie	0.14	73	France
4	Kroemer, Guido	France	10	5527	76	Matson, Vyara	0.04	66	USA
5	Richard, Corentin	France	8	4557	65	Sivan, Ayelet	0.05	62	India
6	Elkrief, Arielle	USA	8	1081	56	Chaput, Nathalie	0.03	42	France
7	Wargo, Jennifer A.	USA	8	3588	49	Viaud, Sophie	0.17	36	USA
8	Messaoudene, Meriem	Canada	7	4395	61	Iida, Noriho	0.08	34	Japan
9	Jenq, Robert R.	USA	6	1245	40	Dubin, Krista	0.03	33	USA
10						Derosa, Lisa	0.02	30	France

TLS, total link strength.

**Figure 5:**
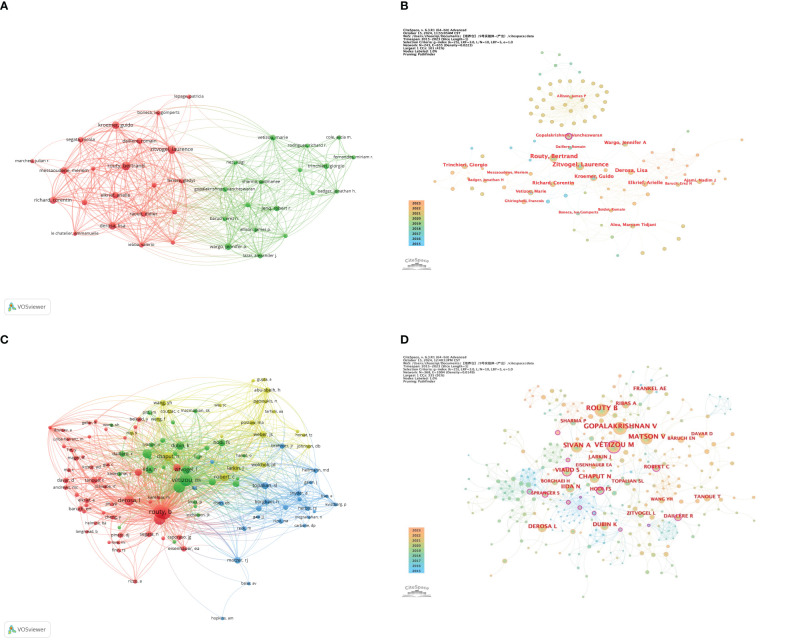
**(A)** Visualization map of co-authorship; **(B)** Centrality map of author collaboration; **(C)** Visualization map of co-citation analyses of authors; **(D)** Map of the centrality of co-citation authors.

**Figure 5A**, constructed with VOSviewer, illustrates the collaboration network of 39 authors who have published at least 3 papers. It is evident that there are two clusters, with the red cluster on the left primarily consisting of French authors and the green cluster on the right mainly comprising American authors. The figure shows that authors within each cluster collaborate closely, while international collaborations between clusters exist but are not dense.

**Figure 5B** is an author collaboration network diagram created with CiteSpace software. The diagram shows that all authors, except for Gopalakrishnan Vancheswaran, have a centrality value not exceeding 0.1, hence only one purple circle appears in the collaboration network diagram. Although this author did not publish more than 5 papers and did not make it into the top nine, their centrality value reached 0.16, indicating that their articles have a certain level of influence.

Further analysis of the co-cited author network (as shown in **Figures 5C, D**) reveals that Routy, Bertrand, Gopalakrishnan, Vancheswaran, and Vétizou, Marie occupy the top three positions in citation frequency with 76, 76, and 73 citations respectively. In this network, Viaud, Sophie and Vétizou, Marie have centrality values exceeding 0.1, at 0.17 and 0.14 respectively, indicating that they play a key role in the research field of gut microbiota and ICIs. The co-cited author collaboration network diagram clearly depicts a close and mature collaboration system, reflecting the strong cooperative relationships that scholars in this field have established.

### 3.6 Top journals analysis

The top 100 most-cited publications in this study are distributed across 58 different academic journals. **Table 5** provides a comprehensive list, showcasing the top 10 journals by publication volume, including key metrics such as the number of papers, country affiliation, impact factor, Journal Citation Reports (JCR) ranking, total citation count, and Total Link Strength (TLS). Most of these journals are affiliated with publishing houses in the United States and the United Kingdom. Among them, “Nature Medicine” leads with 8 papers, followed by “Science” and “Journal for Immunotherapy of Cancer,” which published 7 and 6 papers, respectively.

**Table 5:** Top 10 relevant journals on the application of gut microbiota in the field of ICIs.

Rank	Journal Title	Articles	Country	IF	JCR	Total Citations	TLS
1	Nature Medicine	8	USA	58.7	Q1	1734	84
2	Science	7	USA	44.7	Q1	11274	249
3	Journal for Immunotherapy of Cancer	6	USA	10.3	Q1	694	49
4	Frontiers in Immunology	5	Switzerland	5.7	Q1	785	55
5	Gut	5	England	23	Q1	461	38
6	Annals of Oncology	3	England	56.7	Q1	1596	76
7	Cancer Immunology Research	3	USA	8.1	Q1	452	32
8	Nature	2	England	50.5	Q1	1915	27
9	Nature reviews cancer	2	England	72.5	Q1	1640	36
10	JAMA Oncology	2	USA	22.5	Q1	443	15

TLS, total link strength.

Within these journals, three focus on the field of oncology (“Annals of Oncology,” “Nature Reviews Cancer,” and “JAMA Oncology”), two are dedicated to immunology (“Frontiers in Immunology” and “Cancer Immunology Research”), and in addition, there are two generalist journals (“Science” and “Nature”). All of these journals are located in the Q1 category of JCR, reflecting their high impact and recognition in the academic community.

**Figure 6:**
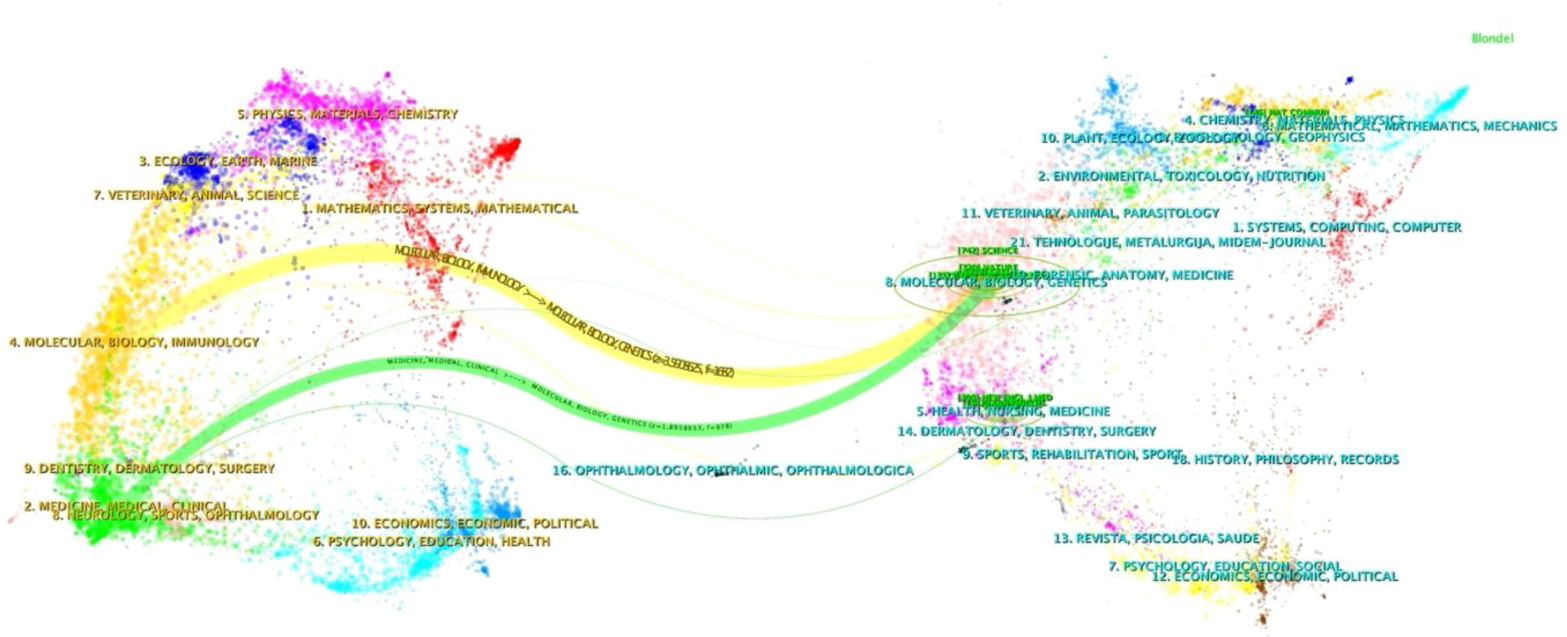
Double-map overlapping journals study intestinal microbiota influe ICIs.

**Figure 6** demonstrates the citation relationships between journals through a double-layer map, revealing two main citation paths. Citing publications are concentrated in disciplines such as molecular biology, biochemistry, immunology, medicine, medical treatment, and clinical practice, while cited publications frequently appear in the fields of molecular biology and genetics. This interdisciplinary citation pattern foretells the breadth and depth of research in the field of gut microbiota and ICIs, indicating the multidisciplinary nature of this area of study and its central position in biomedical research.

### 3.7 Top cited references and co-citation references analysis

In the research on the application of gut microbiota in the field of ICIs, 60 out of the 100 most-cited papers have been cited more than 100 times. According to the data in **Table 6**, the most-cited paper is the study by Routy, Bertrand et al., published in Science in 2018, which explored how the gut microbiome affects the efficacy of PD-1-based cancer immunotherapy (18). The study found that primary resistance to ICIs may be associated with an abnormal composition of the gut microbiome, and the clinical effects may be suppressed in patients treated with ICIs who have used antibiotics. The study has been cited a total of 3,481 times. Following closely is the study by Gopalakrishnan, Vancheswaran et al., which elucidated how the gut microbiome modulates the response of melanoma patients to anti-PD-1 immunotherapy (19). The study found that specific gut microbes, such as *Akkermansia muciniphila*, are associated with a good response to anti-PD-1 therapy. This bacterium can enhance the immune response and improve the effectiveness of immunotherapy, and the study has received 2,956 citations.

**Table 6:** Top 10 Cited Articles on Gut Microbiota in the Field of ICIs.

Rank	Author	Journal	DOI	Year	Citations
1	Routy, Bertrand	Science	10.1126/science.aan3706	2018	3479
2	Gopalakrishnan, Vancheswaran	Science	10.1126/science.aan4236	2018	2596
3	Sivan, Ayelet	Science	10.1126/science.aac4255	2015	2609
4	Havel, Jonathan J.	Nature Reviews Cancer	10.1038/s41568-019-0116-x	2019	1549
5	Cabrita, Rita	Nature	10.1038/s41586-019-1914-8	2020	1210
6	Chaput, Nathalie	Annals of Oncology	10.1093/annonc/mdx108	2017	842
7	Baruch, Erez N.	Science	10.1126/science.abb5920	2021	829
8	Davar, Diwakar	Science	10.1126/science.abf3363	2021	802
9	Pitt, Jonathan M.	Immunity	10.1016/j.immuni.2016.06.001	2016	759
10	Dubin, Krista	Nature Communications	10.1038/ncomms10391	2016	709

**Figure 7:**
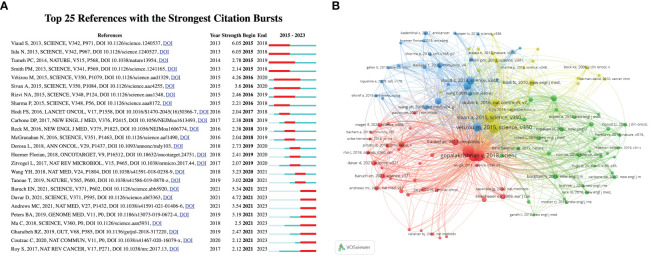
**(A)** Visualization map of top 25 references with the strongest citation bursts from 2015 to 2023; **(B)** The visualization map of references co-citation analysis generated by VOSviewer.

**Figure 7A** further reveals the citation growth trend of the top 25 node papers in this field. The strong citation growth of these papers reflects the current research hotspots. Since 2015, citation activity in this field has begun to rise significantly, with frequent changes in citation hotspots between 2016 and 2020, indicating rapid development in this period. From 2021, a new batch of research hotspots has emerged and continues to the present. This trend indicates that the research combining gut microbiota with ICIs has gained widespread attention and discussion in the academic community, and the heat has continued.

These articles collectively cited 5,451 references, of which 160 papers were frequently cited at least 5 times. Using VOSviewer software for co-citation analysis and visualization (as shown in **Figure 7B**), it was found that four closely related clusters have formed. These clusters focus on applying cutting-edge image processing and machine learning technologies to enhance the detection and classification capabilities of cervical cancer, while emphasizing the importance of automated analysis of cervical cell images for improving the accuracy and efficiency of screening.

The red cluster mainly focuses on the impact of the gut microbiome on the efficacy of ICIs (such as anti-PD-1, anti-CTLA-4). Studies generally believe that the composition and diversity of the gut microbiome are related to the responsiveness to immunotherapy. Some studies have pointed out that specific types of gut bacteria (such as *Akkermansia muciniphila*, *Bifidobacterium* spp., *Faecalibacterium* spp.) are related to anti-tumor immune responses (20, 21). The green cluster mainly focuses on the application of ICIs in the treatment of various types of cancer, especially non-small cell lung cancer (NSCLC), hepatocellular carcinoma (HCC), and melanoma (22–24). Although each study focuses on a specific type of cancer, many findings have been verified in different types of cancer, showing the broad potential of ICIs. The blue cluster emphasizes the correlation between the microbiome and clinical outcomes (such as survival rates, treatment responses, disease progression, and toxicity). Some studies have explored the possibility of improving treatment effects or reducing the toxicity related to immunotherapy and chemotherapy by changing the microbiome (such as using antibiotics, probiotics, or fecal transplantation) (25, 26). The yellow cluster focuses on the connection between the gut microbiome (microbiome) or specific bacteria (such as *Bacteroides genus*, *Clostridium genus*) and cancer treatment (27, 28). Microbiome metabolites, such as short-chain fatty acids (such as butyrate) (29), have been found to regulate immune responses and affect the function and homeostasis of T cells (especially regulatory T cells, Tregs).

### 3.8 Keywords analysis of research hotspots

**Figure 8:**
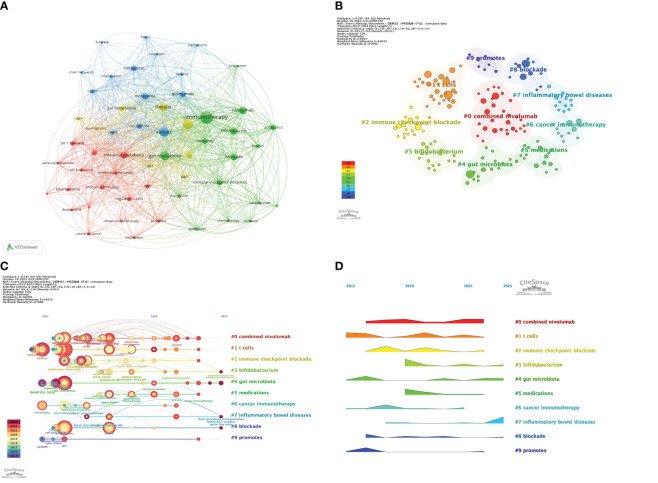
**(A)** Visualization map of keywords generated by VOSviewer; **(B)** Keywords Cluster analysis map; **(C)** CiteSpace visualization map of timeline view; **(D)** CiteSpace visualization map of landscape view. (**Figure 8D** is only used to display an overall trend of popularity and does not directly reflect specific quantitative relationships.).

Keyword analysis reveals the interconnections between research topics and maps out the hotspots and trends within specific research fields. In this study, we conducted an in-depth analysis of 436 keywords and found that 19 keywords were cited more than 10 times. Using VOSviewer software, we constructed a map displaying the co-occurrence of keywords (as shown in **Figure 8A**). In this map, the size of the nodes is proportional to the frequency of the keywords’ appearance, and the thickness of the lines between nodes indicates the strength of their association; the thicker the line, the higher the co-occurrence frequency of the two keywords, and the closer their relationship. Among the many keywords, the node for “immunotherapy” is the largest, followed by “efficacy,” “cancer,” and “gut microbiota.”

Furthermore, we generated a keyword clustering map using CiteSpace software (see **Figure 8B**). The Q value of modularity and the S value of average silhouette are important indicators for evaluating the significance of clustering. The Q value in this study is 0.6994, significantly higher than the benchmark of 0.4, indicating a significant clustering structure; the S value is 0.8555, exceeding 0.5, which suggests that the clustering is efficient and has network homogeneity, with closely related keywords, and the results are convincing. **Figure 8B** shows 10 clusters, and **Table 7** further describes the specific characteristics of each cluster, with “combined nivolumab” and “T cells” being the two largest clusters.

**Table 7:** Details of the top 10 keyword clusters.

Cluster	Size	Silhouette	Year	Top Terms (LLR)
#0 combined nivolumab	30	0.774	2019	combined nivolumab, survival, ipilimumab, bacteria, adenosine
#1 t cells	28	0.761	2017	t cells, immunity, microbiota, efficacy, identification
#2 immune checkpoint blockade	20	0.994	2018	immune checkpoint blockade, adverse events, anti-ctla-4, innate lymphoid cells
#3 bifidobacterium	17	0.906	2019	bifidobacterium, bacteroids fragilis, nanotechnology, akkermansia muciniphila, faecalibacterium prausnitzii
#4 gut microbiota	16	0.965	2019	gut microbiota, checkpoint inhibitor, metabolism, the colonic macrophage, 3d-model
#5 medications	15	0.788	2018	medications, pd-1 antibody, ctla-4 antibody, multicenter, drug resistance
#6 cancer immunotherapy	12	0.903	2017	cancer immunotherapy, clinical response, therapeutic strategies, microbiota-derived metavolites, mhc class i
#7 inflammatory bowel diseases	12	0.944	2021	inflammatory bowel diseases, asaptive immue system, gut microbiota dysbiosis, innate immune system, impact
#8 blockade	11	0.805	2018	blockade, expression, toxicity and iraes, autoimmunity, ici-mediated colitis
#9 promotes	8	0.982	2016	promotes, mait cells, crohns disease, safety, gut

To deeply analyze the keywords in this research field and their development trends, we created a keyword timeline map (**Figure 8C**) and a keyword time peak map (**Figure 8D**). By observing the changes of each cluster over time, a deeper understanding of the key research topics within the field can be gained. **Figure 8C** shows 10 clusters numbered from 0 to 9, with each number indicating the start and end times of the cluster. The size of the nodes reflects the frequency of the keywords’ appearance in the cluster, and the colored lines show the co-occurrence relationships between different clusters. It can be seen that clusters #1, #4, #6, and #9 emerged earlier and continue to receive attention. Current research hotspots include “*Bifidobacterium*,” “gut microbiota,” “inflammatory bowel diseases,” and “blockade.” **Figure 8D** shows that “combined Nivolumab” has been continuously receiving attention since its emergence in 2016 until around 2022. “Gut microbiota” is the earliest emerging cluster and is also the one that has persisted to the present. Continuing popular clusters include “*Bifidobacterium*,” “gut microbiota,” “inflammatory bowel diseases,” and “blockade.” Among them, “inflammatory bowel diseases” have seen a significant increase in popularity in recent years.

## 4 Discussion

Immunotherapy is an advanced treatment method designed to enhance or mimic the natural defense mechanisms of the immune system to combat cancer. Among the various immunotherapy strategies, ICIs play a crucial role. These inhibitors restore the immune system’s ability to recognize and attack tumor cells by blocking the signaling pathways that tumor cells exploit to evade immune surveillance (30). Additionally, the gut microbiota—the collective of all microbes in the human digestive tract—also influences the efficacy of ICIs (31). They modulate the effects of immunotherapy through metabolic byproducts and direct interaction with the host’s immune system (32). This field has become a new frontier in cancer immunotherapy research. Unlike traditional literature review methods, bibliometrics focuses on systematically summarizing the literature in a particular field through quantitative means combined with visualization tools. This method can present complex data in an intuitive way, thereby describing the progress patterns of the research field and predicting future research directions.

This study attempts to use bibliometric methods to summarize the application of gut microbiota in the field of ICIs, and for the first time, it analyzes in detail the top 100 most-cited papers in this field. We used three bibliometric tools: CiteSpace, VOSviewer, and Bibliometrix R package, leveraging their powerful visualization capabilities to deeply demonstrate current research hotspots and potential future development trends. Through this approach, we not only explored the applications and development trends of this field but also identified current research hotspots and made reasonable predictions for potential future research directions.

By reviewing the research on the application of gut microbiota in the field of ICIs, we analyzed the total number of citations and the annual distribution of the top 100 cited papers to observe the evolutionary trend of the role of gut microbiota in immune checkpoint inhibitor research in terms of quantity and quality. In the early stages of the research, the number of included articles was relatively low, but the average citation frequency was very high (for example, only one paper was included in 2015, but its citation frequency reached 2588 times), indicating that early research papers played a foundational role in the entire field of study. Subsequently, the average number of citations showed a trend of increasing and then decreasing, with the total number of citations peaking in 2018, reflecting a very active year in the field. The decline in citation frequency was largely influenced by time, as the accumulation of citations is strongly related to the passage of time. As time goes on, newly published articles require time to accumulate citations, which may result in relatively lower citation frequencies for articles published in recent years. This trend reflects the fluctuations in the popularity of research in this field and also indicates the high recognition and reliance of researchers on early research outcomes.

Among the top 100 most-cited articles, the United States leads globally in contributions, with its published research spanning from 2015 to 2023 (as shown in **Figure 3B**), holding an advantage in terms of publication volume, total citation frequency, and total link strength. However, in terms of centrality ranking, the United States is only in the seventh position, lower than Italy and France (with a centrality value of 0.33). This phenomenon indicates that although the United States has outstanding performance in the number of publications and citation frequency, there is still room for improvement in international collaboration.

According to the data in **Table 2**, the United Kingdom tops the list with an extremely high centrality value of 1.24, despite having only 8 included publications. This data highlights the close cooperation of the United Kingdom with the global research community and the significant impact of its research results worldwide. The high centrality value of the United Kingdom reflects its central position in the international scientific collaboration network and the notable contributions its research has made to the global scientific community. These findings emphasize the importance of international cooperation in driving scientific development and the different roles that various countries play within the global research network.

In the field of tumor immunotherapy, the research advancements in France and Germany are noteworthy. French research has focused on exploring how the gut microbiome affects the efficacy of ICIs, such as by analyzing the relationship between specific bacterial species and patients’ responses to ICIs. Germany has made significant progress in the study of the tumor immune microenvironment, particularly in understanding the mechanisms of interaction between immune cells and microbes within the tumor microenvironment. These studies not only enhance our understanding of the role of microbes in tumor immunotherapy but also provide a scientific basis for the development of new treatment strategies.

To further promote international cooperation, the collaborative models in tumor immunotherapy between France and Germany can be emulated. In France, biotechnology company OSE Immunotherapeutics has partnered with Nantes University Hospital to conduct clinical trials aimed at evaluating the effectiveness of new cancer immunotherapy approaches. This collaboration provides researchers with a unique platform to explore and develop more effective treatment methods. In Germany, Merck KGaA and Pfizer have jointly developed and promoted the PD-L1 immune checkpoint inhibitor Bavencio (avelumab), and have tested its effects in combination with other anticancer therapies in numerous clinical trials. These cooperation models demonstrate the importance of international collaboration in advancing cancer immunotherapy research and clinical applications, providing valuable experience and models for scientific cooperation worldwide.

The work of research institutions is often influenced by the country they belong to, which is particularly evident among the top 10 institutions with the highest publication volume included in the top 100 studies. These institutions highlight their significant value in the field. According to the data in **Table 3**, 8 of these institutions are from France, which not only reflects the increasingly prominent position of France in the research field of applying gut microbiota to ICIs but also shows that France is gradually becoming an important center for this research theme.

Centrality is an important indicator for assessing the strength of cooperation between countries. Observing the centrality of French institutions, we find that they remain higher than those of other countries, indicating that French institutions have a strong influence in the field and that the research activity in France is more intense than in other countries. The high centrality value of French institutions indicates that they play a core role in the international scientific cooperation network, and their research outcomes are not only valued domestically but also have a broad impact globally. These data emphasize France’s significant contribution to the global field of gut microbiota and immune checkpoint inhibitor research, as well as its important role in promoting scientific progress in this field.

By conducting an in-depth analysis of the author collaboration network, we observed, as shown in **Figure 5B**, that instances where an author’s centrality exceeds 0.1 are not common, but they do exist. This indicates that while there is a certain level of cooperation among authors, an extensive international collaboration network has not yet been formed. This is also reflected in the node distribution in **Figure 5A**. In **Figure 5A**, two distinct clusters can be clearly identified, with authors within these clusters working closely together, while cooperation between clusters is relatively sparse. Although most of the authors ranked in the top 9 for publication volume are from France, the author with the highest total citation volume is Routy, Bertrand, from Canada. His research focuses primarily on exploring the intrinsic and extrinsic factors that contribute to cancer cells’ resistance to ICIs and how to overcome these resistances (33). Additionally, he is dedicated to studying how to use information from the gut microbiome to develop new cancer treatment strategies, including Fecal Microbiota Transplantation (FMT) and the supplementation of probiotics/prebiotics (34, 35). The work of Routy, Bertrand has not only had a profound impact on the academic community but has also brought new perspectives and potential therapeutic avenues to the field of cancer treatment.

In the co-citation analysis of authors, Routy, Bertrand still ranks first in citations, even though his centrality value did not reach 0.1 and the node was not marked with a purple circle in **Figure 5D**. His leading position is largely attributed to a study published in Science magazine in 2018 on the relationship between *Akkermansia muciniphila* and the efficacy of PD-1 blockade therapy (18). The study found that, through metagenomic analysis of patients’ fecal samples, *Akkermansia muciniphila* was relatively more abundant in patients who responded to PD-1 blockade therapy. The study also found that oral supplementation of *Akkermansia muciniphila* could restore the efficacy of PD-1 blockade after antibiotic treatment, an effect that depends on IL-12 and is mediated by increasing the infiltration of CCR9+CXCR3+CD4+ T lymphocytes into the tumor bed of mice. This article has been cited 3,479 times. Among the top 10 authors ranked by co-citation frequency, Viaud, Sophie has the highest centrality value, reaching 0.17. His research focuses on exploring how cyclophosphamide alters the composition of the small intestine microbiota, prompting specific bacterial species to migrate to secondary lymphoid organs, thereby stimulating specific T cell subsets and enhancing anti-cancer immune responses (36, 37). Additionally, Viaud, Sophie is dedicated to studying the immunosuppressive role of IL-18 in cancer and how anti-PD-1 antibodies can exert clinical effects in human malignant tumors (38, 39). These studies not only deepen our understanding of the interaction between the gut microbiota and anti-cancer immune responses but also provide important scientific evidence for the development of new cancer treatment strategies.

In the interdisciplinary field of gut microbiota and ICIs research, according to the data in **Table 5**, “Nature Medicine” (IF=58.7, Q1), “Science” (IF=44.7, Q1), and “Journal for Immunotherapy of Cancer” (IF=10.3, Q1) are the top three journals in terms of the number of articles included in the top 100 most-cited papers in this field. Impact Factor (IF), Journal Citation Reports (JCR) classification, total citation volume, and Total Link Strength (TLS) are key indicators for measuring the academic level of journals. “Science” has the highest total number of citations among the top 10 journals by publication volume, reaching 11,274 times, indicating its significant position in this academic field. These data imply that these journals may prioritize publishing more research papers on how gut microbiota affect the efficacy of ICIs in the future. The high impact and academic recognition of these journals provide an important publishing platform for research in the field of gut microbiota and ICIs.

Journals such as “Nature Medicine,” “Science,” and “Journal for Immunotherapy of Cancer,” with their high publication volumes, are expected to continue publishing high-quality research outcomes, making significant contributions to the advancement of the field of gut microbiota and ICIs. The citation patterns of papers in these journals reveal a phenomenon: the cited literature is primarily concentrated in the fields of molecular biology and genetics, while citing papers broadly cover various fields including molecular biology, biochemistry, immunology, medical treatment, and clinical practice. This reflects the depth and breadth of interdisciplinary research in the cross-field of gut microbiota and ICIs, indicating the field’s reliance on a substantial foundation and accumulation of basic research. This interdisciplinary research trend not only demonstrates the complexity of the research in this field but also shows the extent of its widespread attention from multiple perspectives.

By examining the top 10 highly cited papers related to the application of gut microbiota in the field of ICIs, we can gain insights into the research hotspots and trends in this field. These papers typically focus on how the gut microbiome affects the therapeutic response of cancer patients to ICIs (such as PD-1/PD-L1 inhibitors), including analyzing the differences in gut microbiome composition between patients who respond and do not respond to immunotherapy, how the gut microbiome regulates the host’s immune system, and how this regulation affects anti-tumor immune responses. In **Table 6**, the most cited paper is from Routy, Bertrand et al., whose contributions have been detailed in the aforementioned section on authors. The paper by Gopalakrishnan, Vancheswaran et al., published in Science in 2018, received 2956 citations and ranks second (19). This study mainly explored the impact of gut microbiota composition on the cancer immunotherapy of patients with renal cell carcinoma (RCC), finding that the composition of the gut microbiota in patients treated with immune checkpoint blockade (ICB) was influenced by antibiotics (ATBs) and tyrosine kinase inhibitors (TKIs). In the RCC mouse model, primary resistance to ICB could be overcome by fecal microbiota transplantation (FMT) or by administering specific symbiotic bacteria. The study emphasizes the importance of gut microbial communities in cancer treatment responses and suggests the potential for improving the efficacy of cancer immunotherapies by modulating the gut microbiota, providing new ideas for future research directions and clinical applications.

Keyword analysis provides us with a unique perspective to understand the development trajectory and trends of gut microbiota in the field of ICIs. To comprehensively grasp the hotspots and cutting-edge topics in this field, we utilized VOSviewer software for a visual analysis of high-frequency keywords, as shown in **Figure 8A**. The analysis revealed keywords such as “immunotherapy,” “efficacy,” “cancer,” and “gut microbiota,” which reflect the current research focus. Currently, research on gut microbiota in the field of ICIs mainly concentrates on several aspects: exploring the impact of gut microbiota on treatment outcomes, managing side effects, gaining a deeper understanding of mechanisms of action, and developing gut microbiota-based interventions and biomarkers. Among these, fecal microbiota transplantation (FMT) and antibiotic treatment are the most widely used techniques. These studies not only advance our understanding of the complex interactions between gut microbiota and cancer treatment but also provide new directions for developing new therapeutic strategies and enhancing the efficacy of existing therapies. Through this analysis, we can more clearly see the importance and future development potential of gut microbiota research in the field of ICIs.

By conducting an in-depth analysis with CiteSpace software, as shown in **Figure 8B**, we performed keyword clustering and found that “#0 combined nivolumab” and “#1 T cells” are two of the largest clusters. This discovery signifies that research in this field is moving towards a multidimensional and comprehensive direction, emphasizing the key role of the microbiome in regulating immune responses and optimizing therapeutic outcomes. Nivolumab, as a PD-1 inhibitor, has been widely used in the immunotherapy of various cancers. Its combined use with other treatment modalities (such as chemotherapy or other ICIs) has shown enhanced therapeutic effects (40, 41). This indicates that researchers are actively exploring how to improve the effectiveness of immunotherapy through combination therapies. At the same time, T cells play a central role in anti-tumor immune responses. Studies have shown that the gut microbiota can influence the efficacy of ICIs by modulating the function and activity of T cells. For example, specific gut bacteria, such as *Bifidobacterium* and *Akkermansia muciniphila*, can promote the activation and proliferation of T cells, thereby enhancing the immune response to tumors (18, 19, 42, 43).

The timeline view analysis (**Figure 8C**) further reveals the trend of clinical applications of gut microbiota research in the field of artificial intelligence. With the in-depth exploration of the gut microbiome, the focus of research has gradually shifted from basic theory to the exploration of impacts on treatment outcomes, management of side effects, in-depth understanding of mechanisms of action, and the development of interventions and biomarkers based on the gut microbiota. Building on early theoretical research and technological exploration, research on gut microbiota has permeated various fields of cancer immunotherapy, including the modulation of tumor and immune cell functions (18, 27, 42), promotion of the production of various cytokines (44), prediction of responses to ICIs (45, 46), and enhancement of the efficacy of ICIs (47, 48). These studies have yielded encouraging results, providing important guidance and support for clinical treatment decisions and improvement of therapeutic outcomes.

**Figure 9:**
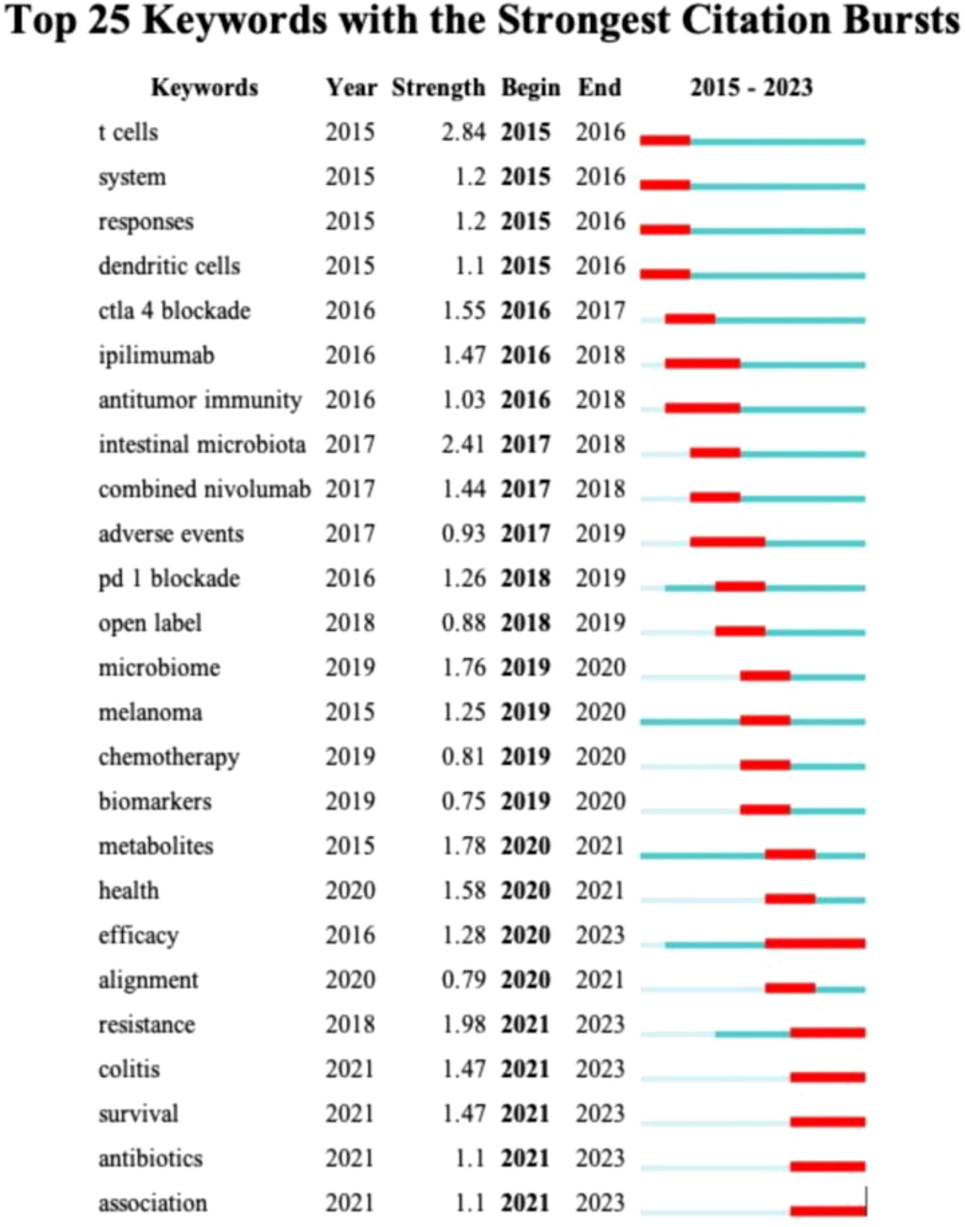
Visualization map of top 25 keywords with the strongest citation bursts from 2015 to 2023.

Keyword emergence analysis (**Figure 9**) reveals a relatively flat trend in the evolution of keywords from 2016 to 2020. During this period, the research focus gradually shifted from individual cell types or single blocking methods to the study of a class of diseases and the discussion of combined treatment plans. For instance, the transition from keywords such as “ctla 4 blockade” and “ipilimumab” in 2016 to “health,” “efficacy,” and “alignment” in 2020 indicates that the research perspective is shifting from a microscopic level to a more macroscopic treatment strategy. Entering the year 2020, the research field ushered in a period of rapid development, with research priorities beginning to focus on the management of colitis induced by ICIs, the improvement of patient survival rates, and the impact of antibiotics on gut microbiota and immune treatment effects. These changes not only reflect the deepening and expansion of research content but also indicate the scientific community’s emphasis on the side effects related to immunotherapy and treatment optimization.

Overall, the application of gut microbiota in the field of ICIs can be summarized into five main research directions. Firstly, the composition and diversity of the gut microbiota have a crucial impact on the efficacy of ICIs. Taiki Hakozaki and his colleagues found that the use of antibiotics in patients with non-small cell lung cancer (NSCLC) reduces the effectiveness of ICIs, which is associated with the enrichment of *Ruminococcaceae UCG 13* and *Agathobacter* in the gut (48). N. Jan and others pointed out that fecal microbiota transplantation (FMT) can increase the expression of IL-25, a key immune modulator that promotes the differentiation of Th2 cells and suppresses inflammatory responses (49). In addition, Jindong Zhang and his colleagues discovered that FMT can also affect the activity and function of immune cells by altering the functional genes and metabolic products of gut microbes (50). These findings not only reveal the complex relationship between gut microbiota and the efficacy of ICIs but also provide new ideas for developing new treatment strategies and improving the effectiveness of existing therapies.

Secondly, the composition of the gut microbiota is directly related to the effects of ICIs and the occurrence of immune-related adverse events (irAEs). Research by Zhaozhen Wu found that in patients receiving both anti-PD-1 and chemotherapy, different gut microbes were associated with the occurrence of adverse events (AEs) (51). Bacteria from the Bacteroidetes phylum, particularly the *Bacteroides genus*, were more abundant in patients who did not experience AEs, while bacteria from the Firmicutes phylum, such as *Faecalibacterium prausnitzii*, were more abundant in patients who did experience AEs. Chaput, Nathalie’s study showed that patients who responded to treatment had a richer presence of beneficial bacteria in their gut (52), such as *Akkermansia muciniphila* and *Faecalibacterium*. Yifan Zhang’s research found that the occurrence of irAEs is associated with specific gut microbiota and their metabolic activities (53). These findings suggest that adjusting the gut microbiota may improve the therapeutic effects of ICIs and reduce adverse events.

Thirdly, investigating how the gut microbiota affects the mechanism of action of ICIs is extremely crucial. Research by Yuting Lu and colleagues suggests that specific gut microbes and their metabolites may interact with innate and/or adaptive immune cells, altering the tumor microenvironment (TME) and thereby affecting the efficacy of ICIs (54). Feng Wang has pointed out that *Bifidobacterium*, by adjusting the composition of the gut microbiota, can systematically affect the function of regulatory T cells (Treg), thereby improving intestinal immunopathology and enhancing anti-melanoma immunity (55). These findings emphasize the potential regulatory role of the gut microbiota in the efficacy of ICIs and reveal new avenues that might optimize immunotherapy by modulating gut microbes.

Fourthly, interventions based on the gut microbiota have shown significant effects in practice. Research by Derosa, Lisa and colleagues indicates that gut microbes are not only involved in the absorption and metabolism of nutrients but also affect the host’s immune system by producing metabolic products such as short-chain fatty acids(SCFAs) (56). These metabolic products can regulate the function of immune cells, including T cells and dendritic cells, thereby affecting the efficacy of ICIs. Different compositions and functions of gut microbiota can lead to the production of different types and quantities of SCFAs (57). Studies have shown that the gut microbiota is closely related to the production of SCFAs (58). Germ-free mice, which lack gut microbiota, have very low concentrations of SCFAs in their intestines and peripheral tissues, further confirming the close relationship between the gut microbiota and the production of SCFAs. Grant Wilson and others emphasized the importance of intestinal barrier integrity, considering it crucial for preventing pathogens and toxins from entering the bloodstream (59). The use of antibiotics may affect the tumor microenvironment and the function of immune cells by disrupting the intestinal barrier and increasing inflammatory responses.

Lastly, the gut microbiota shows great potential as a biomarker for predicting the efficacy of ICIs. Utilizing precise microbiome analysis platforms, researchers have been able to develop highly accurate biomarkers to predict the effects of ICIs. For instance, Mat Robinson and colleagues used the Microbiotica platform and machine learning models to validate the effectiveness of specific microbial signatures in multiple independent studies (60). To further explore the potential of the gut microbiota as a biomarker, large-scale prospective clinical trials are currently underway. Philippa Gail Corrie and others are planning to recruit up to 1800 patients receiving ICI treatment, aiming to explore and validate the predictive value of gut microbiota signatures across various cancer types (61). These research advancements not only highlight the application prospects of the gut microbiota in predicting the efficacy of ICIs but also provide new directions for personalized medicine.

The application and integration of the gut microbiome in the field of immune checkpoint inhibitors (ICIs) signifies the birth of a new interdisciplinary research system. This system shows tremendous potential and value in terms of therapeutic outcomes, side effect management, exploration of mechanisms of action, intervention strategies, and the development of biomarkers. Studies have demonstrated that the gut microbiome plays a crucial role in the treatment of immune checkpoint inhibitors and provides a new perspective and potential therapeutic strategies for cancer treatment. In particular, certain gut bacteria such as *Bifidobacterium*, Akkermansia, and Bacteroides have been considered to effectively enhance antitumor immunity and control the growth of tumors within the body. Moreover, metabolites of the gut microbiome, such as inosine, short-chain fatty acids, and ursolic acid, can diffuse from the gut and affect both local and systemic antitumor immune responses, thereby improving the efficacy of ICIs.

Looking ahead, strengthening interdisciplinary collaboration in the fields of microbiology, immunology, oncology, and emerging technologies such as computer science will help us fully understand the mechanisms of action of the gut microbiota in immunotherapy. With technological advancements and the development of new therapies, we can use gene-editing technologies such as CRISPR to develop new intervention strategies for the gut microbiome and explore the application of microbial therapies in cancer treatment. Additionally, personalized treatment considering the gut microbiome will become a focus of research. By tailoring treatment plans based on the characteristics of a patient’s gut microbiome, we can achieve more precise and effective treatments. This personalized approach not only enhances the efficacy of ICIs but also reduces side effects, thus advancing the application of precision medicine with the microbiome. By manipulating the gut microbiome (such as fecal microbiota transplantation (FMT), probiotics, engineered microbes, and specific microbial metabolites), we can develop rational treatment strategies based on the microbiome, bringing more personalized and effective treatment plans for cancer patients, ultimately improving their quality of life and survival rates. Emerging technologies such as artificial intelligence and machine learning also play an important role in enhancing the gut microbiota in ICIs research. Machine learning algorithms have been proven to be useful in identifying key molecular characteristics, discovering potential patient stratification, and generating models that can accurately predict phenotypes (62). In the multi-omics data analysis of the gut microbiome, machine learning algorithms can integrate different types of molecular profiling data, such as metagenomics, metatranscriptomics, and metabolomics, to provide more comprehensive analysis results (63). Additionally, machine learning methods can be used to develop gut microbiome-targeted therapies, contributing to the realization of personalized and precision medicine (62). The application of these technologies not only improves the efficiency and accuracy of research but also offers new possibilities for the development of new therapeutic strategies and interventions.

Despite the significant progress and great potential shown by the gut microbiota in the field of immune checkpoint inhibitor (ICI) research, there are still challenges and limitations to face in practical applications. The main challenge is the issue of individual variability. The composition of the human gut microbiota is influenced by various factors such as diet, lifestyle, geography, and ethnicity, showing great heterogeneity (64). This variability poses a problem for defining universal microbiome-based biomarkers, as the gut microbiota composition can significantly differ among different populations. Moreover, the interaction between the gut microbiota and the host is also affected by genetic and environmental factors (65), further increasing the complexity of individual differences.

Although these characteristics of the gut microbiota provide opportunities for personalized medicine, accurately identifying and utilizing these differences in practical applications remains a challenge. For instance, while studies suggest that the composition of the gut microbiota may affect a patient’s response to ICI therapy (66), the highly heterogeneous composition of the gut microbiota means that microbes identified from feces may only be markers for other factors that are associated with immune responses and have a connection with the microbiota. Additionally, although studies have demonstrated the impact of microbiota on the effects of ICIs in germ-free mouse models through fecal transplantation, species-specific biological differences prevent direct extrapolation of results from animal models to human models (67).

Therefore, while the gut microbiota holds great potential in ICI research, overcoming the challenge of individual variability requires a more personalized approach to modulating the microbiome. This may include developing comprehensive models (68) that consider the host’s genetic background, lifestyle, and environmental factors, as well as utilizing deep metagenomic sequencing and big data analysis to better understand the diversity and function of the gut microbiota (69).

## 5 Advantages and limitations

Our study has some notable limitations. Firstly, the literature search primarily relied on the SCI-E database within the Web of Science Core Collection. Although we carefully selected indices closely related to the research topic, this selection may have omitted relevant and important literature from other databases, potentially introducing selection bias. Additionally, due to the limitations of bibliometric software and platform algorithms, some degree of data screening and integration was inevitably performed during the parameter setting process, which may have led to certain systematic errors, although we have strived to minimize such errors. Lastly, since the accumulation of citation counts takes time, our study may not fully reflect the impact of recently published literature, which to some extent also affects the accuracy of the assessment results.

Additionally, we would like to provide a special note regarding the image issue. Since the images are automatically generated by the software platform and subject to layout constraints, we are unable to standardize the format of project names (such as researchers’ names). However, these differences in formatting do not affect the scientific information and accuracy of the data conveyed by the charts. We ensure the core content and precision of the data in the charts so that readers can accurately understand the research findings presented in the images.

Despite these limitations, our study remains committed to selecting the most representative existing literature regarding the application of the gut microbiota in the field of ICIs, and provides valuable insights into the research hotspots, trends, and future development directions of this field. We believe that, despite these restrictions, our research findings can still offer profound insights to the academic community and guide future research.

## 6 Conclusion

This study employs bibliometric methods to conduct an in-depth analysis of publications related to the application of gut microbiota in the field of ICIs. The analysis reveals the pivotal roles played by the gut microbiota in the development of ICIs, including influencing treatment efficacy, managing side effects, elucidating mechanisms of action, devising intervention strategies, and developing biomarkers. Currently, the research focus in this field is gradually shifting towards addressing side effects related to immunotherapy, with the aim of optimizing treatment strategies.

It is particularly noteworthy that China and the United States have achieved representative results in this research field and are expected to continue their leading positions in the future. To further advance the development of this field, strengthening international exchange and cooperation is particularly urgent. Establishing closer collaborative relationships with scientific powerhouses such as the United States and France, or countries with strong research presence in this field, will facilitate the exchange of knowledge and joint technological progress.

Additionally, interdisciplinary collaboration is particularly important in advancing research on the gut microbiome. By pooling the expertise of specialists in computer science, bioinformatics, microbiology, and clinical medicine, we can develop advanced data analysis models that not only guide the design of personalized cancer immunotherapy treatment plans but also improve patient response rates and therapeutic outcomes to ICIs. This collaborative approach fosters a deeper understanding of the complex interactions between the gut microbiome and cancer treatment, especially in areas that are not yet fully explored, such as the role of the gut microbiome in rare types of cancer and the impact of specific microbial communities on the response to immunotherapy. In-depth research in these areas will provide clearer directions for future studies, helping us to develop more precise treatment strategies. Such interdisciplinary efforts not only accelerate scientific discoveries but also have the potential to revolutionize the prospects of cancer treatment, offering patients more personalized and effective treatment options, ultimately improving their quality of life and survival rates.

## Data Availability

The original contributions presented in the study are included in the article/**Supplementary Material**. Further inquiries can be directed to the corresponding author.
