# Supplementary figures and images for: A bibliometric study on the impact of gut microbiota on the efficacy of immune checkpoint inhibitors in cancer patients: analysis of the top 100 cited articles
Source: Front Immunol. 2025 Jan 16;15:1519498. doi: 10.3389/fimmu.2024.1519498 (PMC11779710; doi:10.3389/fimmu.2024.1519498)

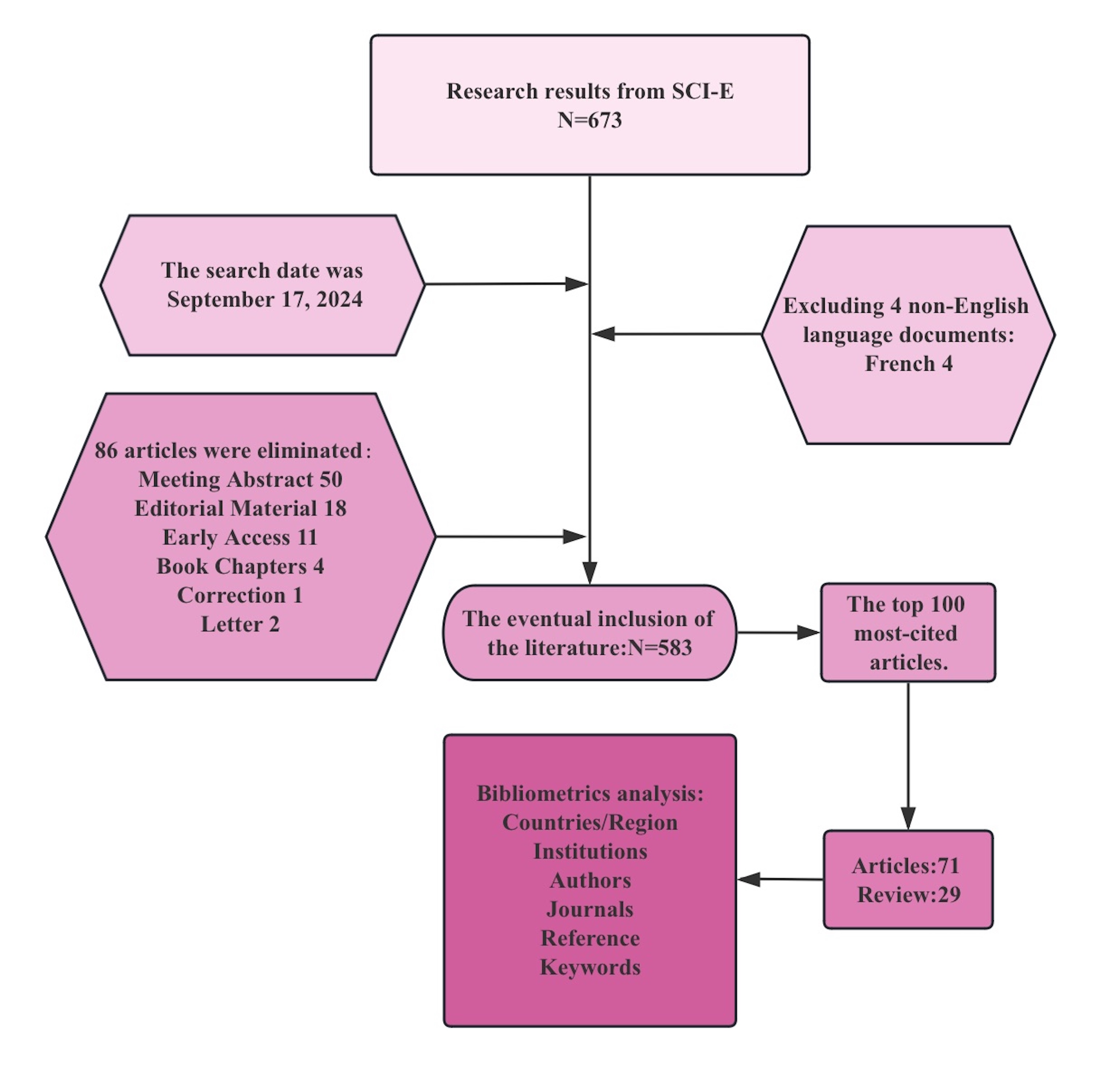

Supplement: Supplementary file 1 [file DataSheet1.zip › Supplementary Material Presentation/Figure1.jpeg]

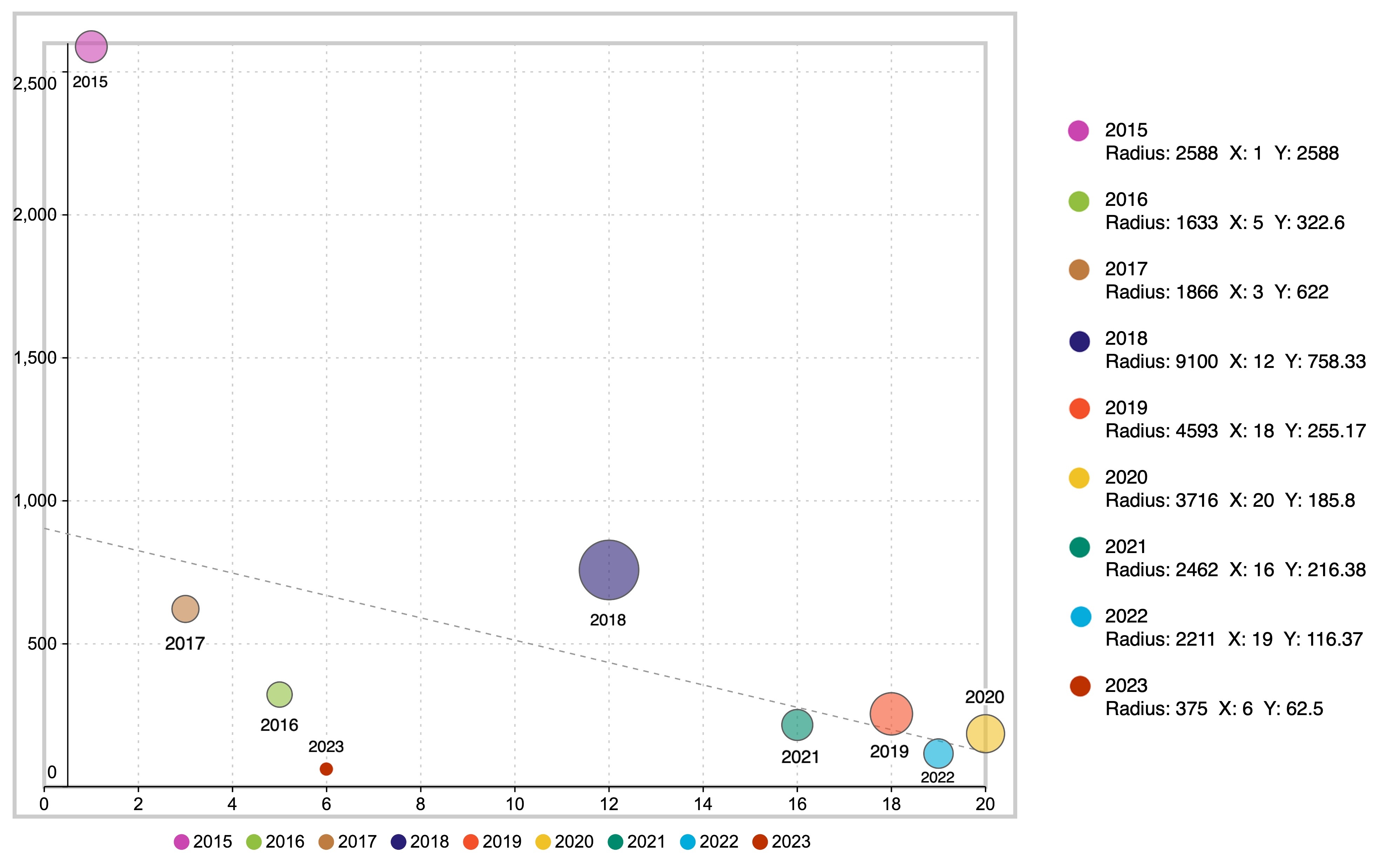

Supplement: Supplementary file 1 [file DataSheet1.zip › Supplementary Material Presentation/Figure2.jpg]

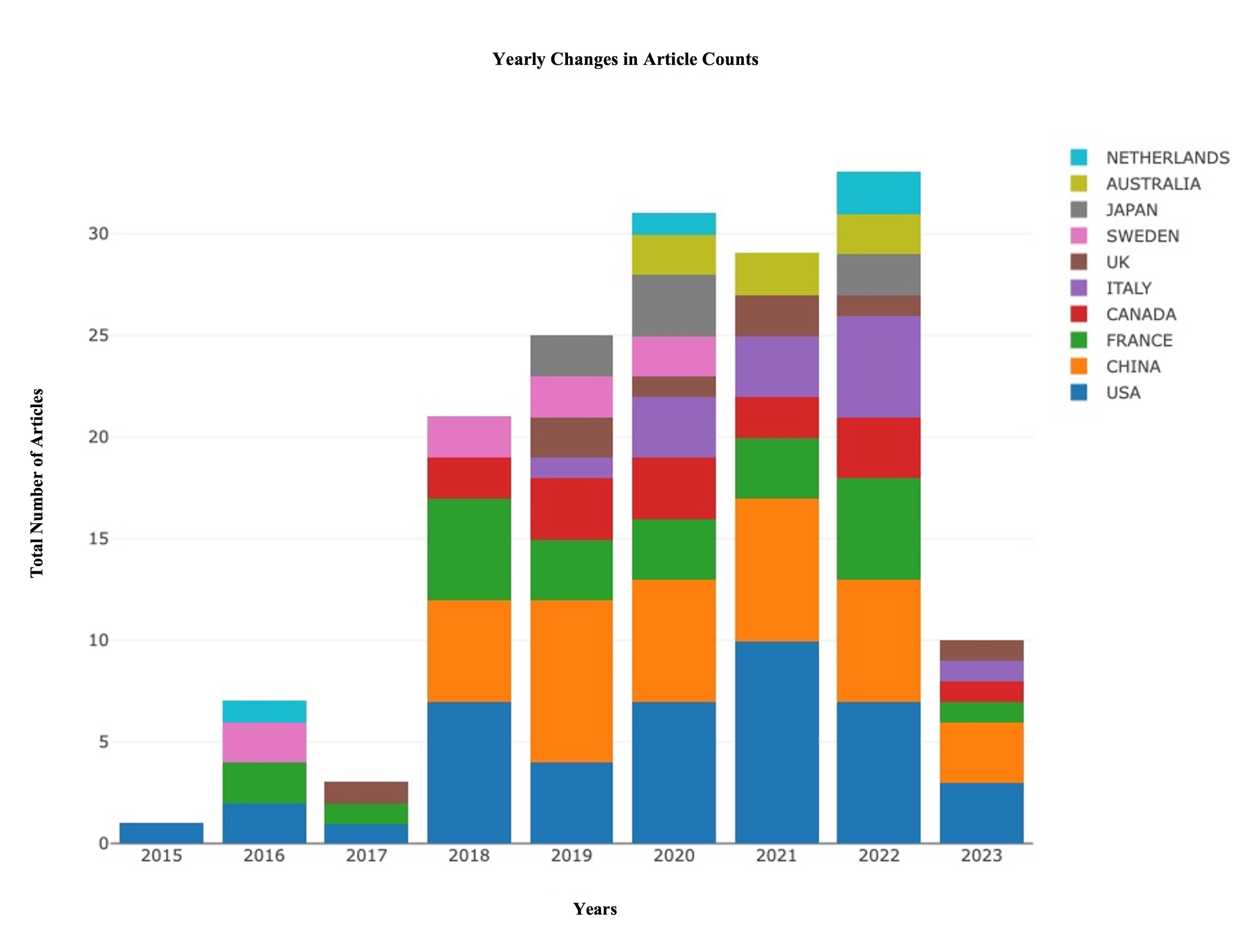

Supplement: Supplementary file 1 [file DataSheet1.zip › Supplementary Material Presentation/Figure3B.jpeg]

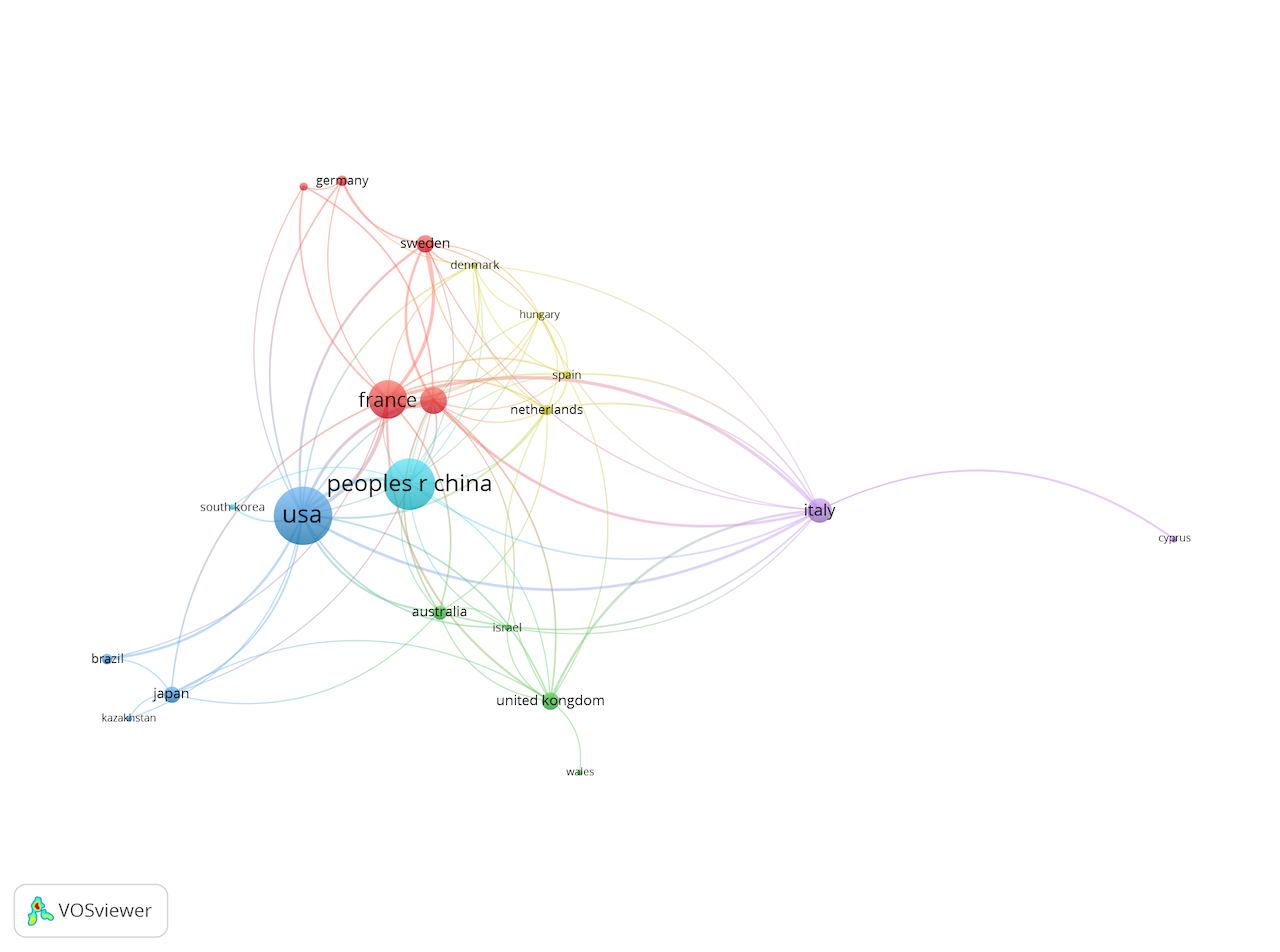

Supplement: Supplementary file 1 [file DataSheet1.zip › Supplementary Material Presentation/Figure3C.tiff]

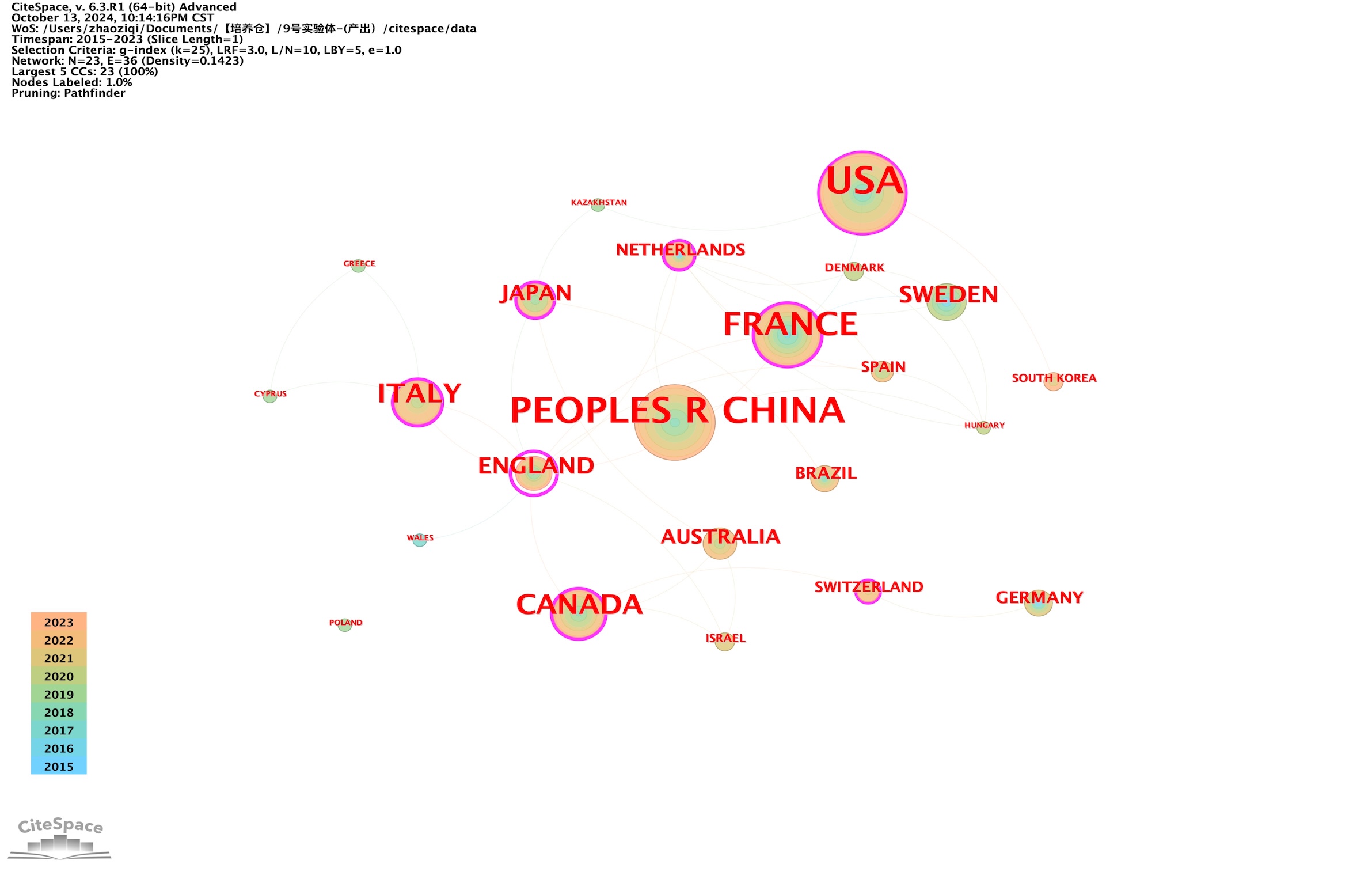

Supplement: Supplementary file 1 [file DataSheet1.zip › Supplementary Material Presentation/Figure3D.jpeg]

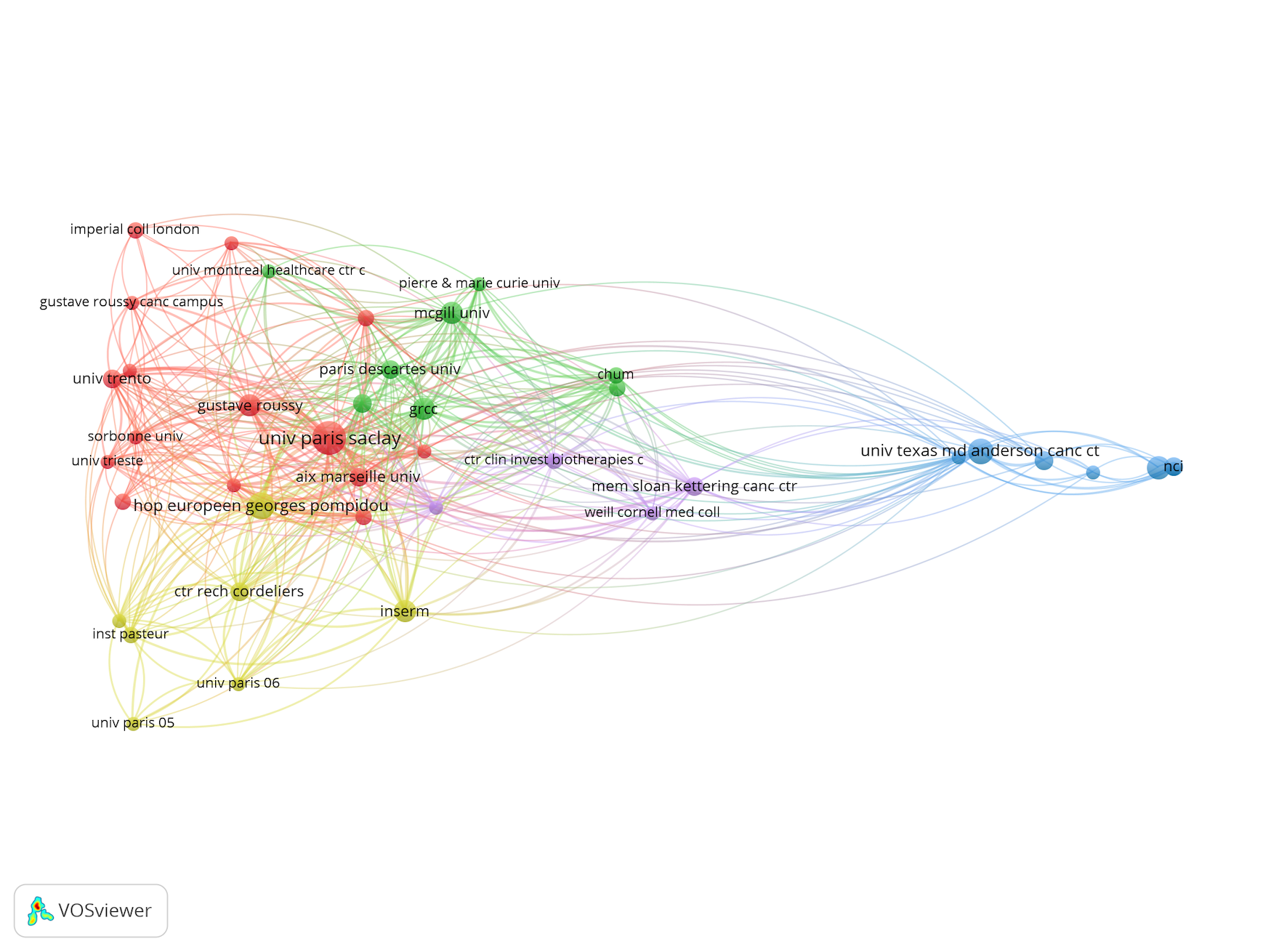

Supplement: Supplementary file 1 [file DataSheet1.zip › Supplementary Material Presentation/Figure4.tiff]

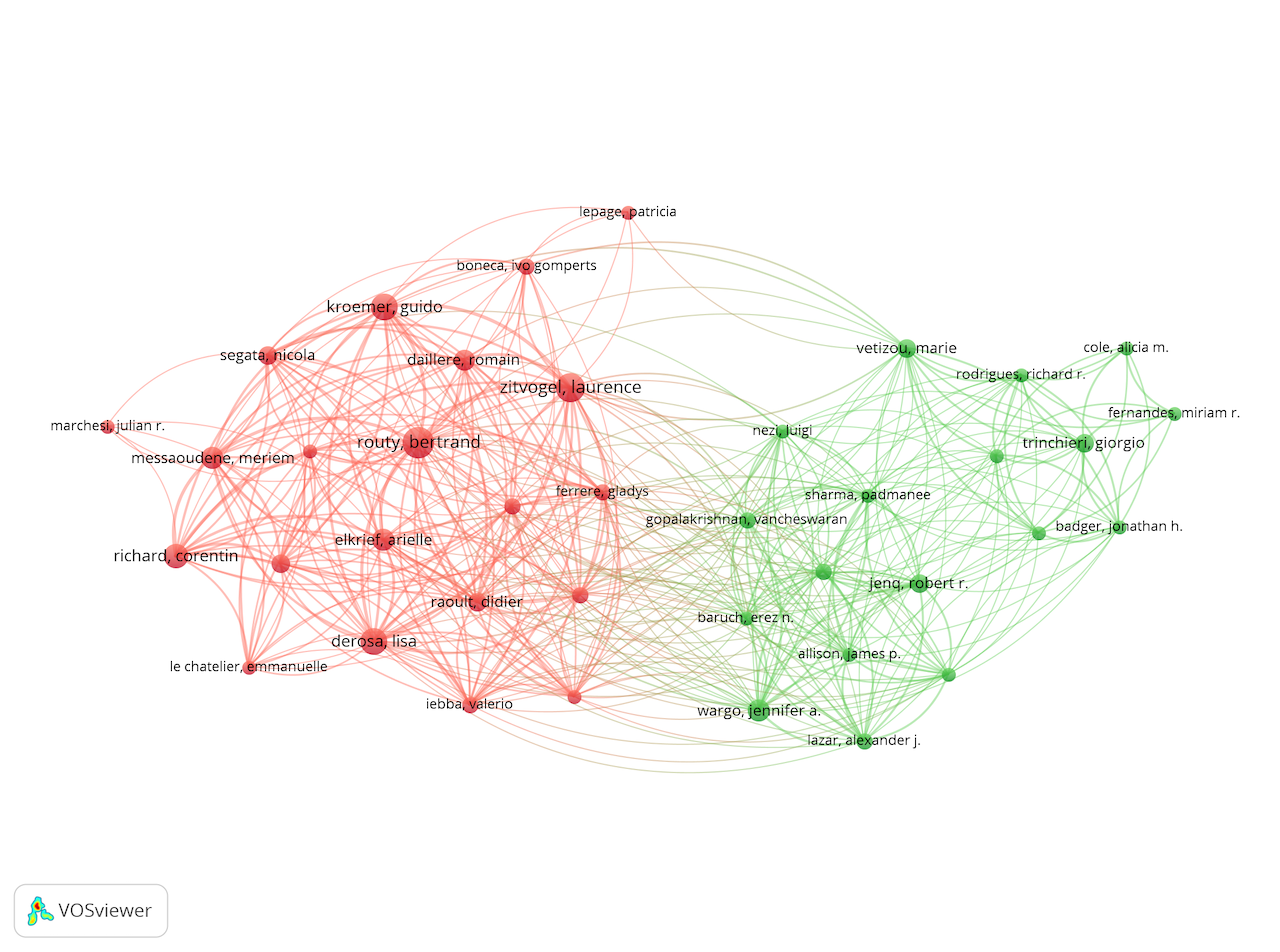

Supplement: Supplementary file 1 [file DataSheet1.zip › Supplementary Material Presentation/Figure5A.tiff]

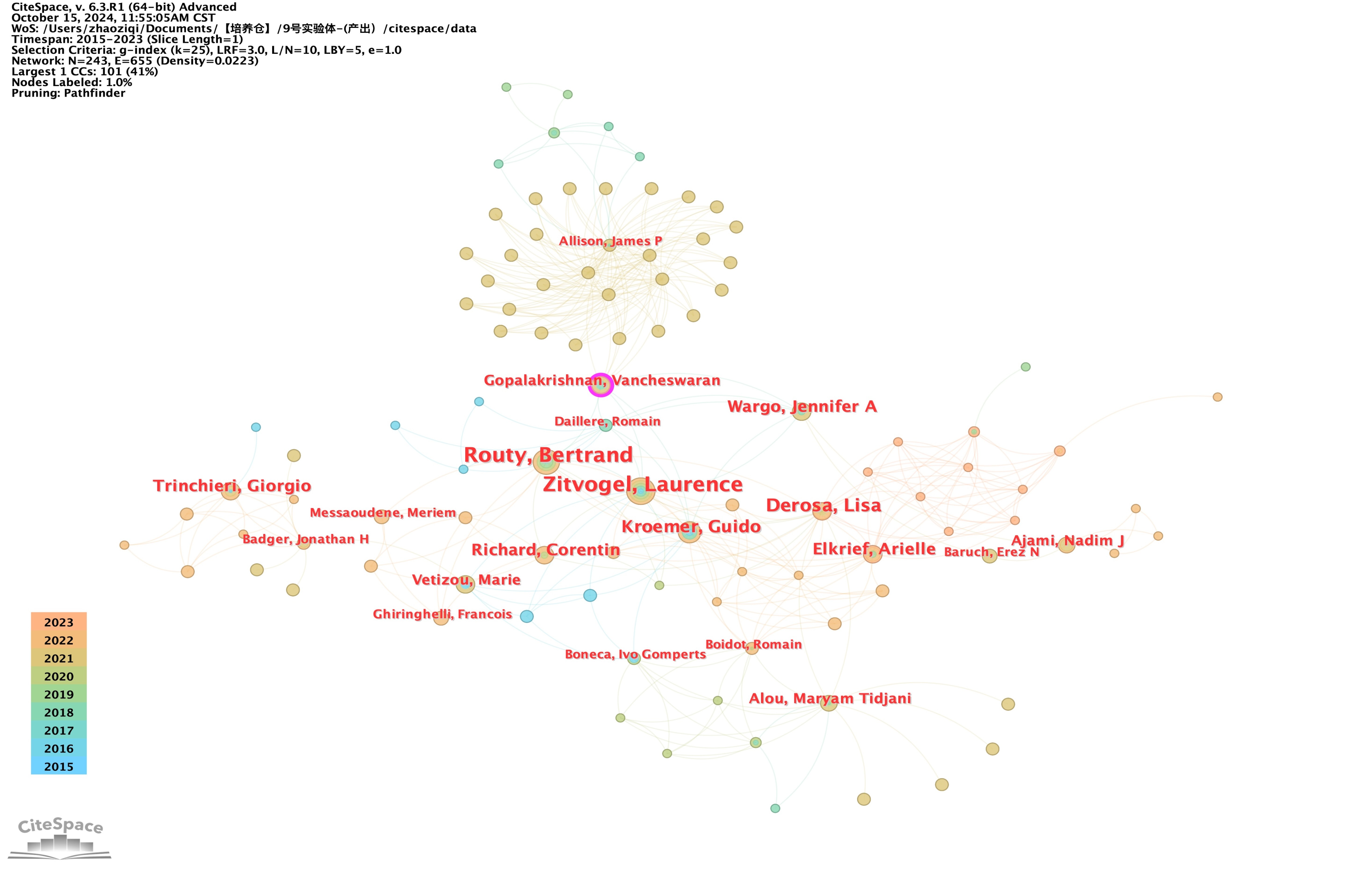

Supplement: Supplementary file 1 [file DataSheet1.zip › Supplementary Material Presentation/Figure5B.jpeg]

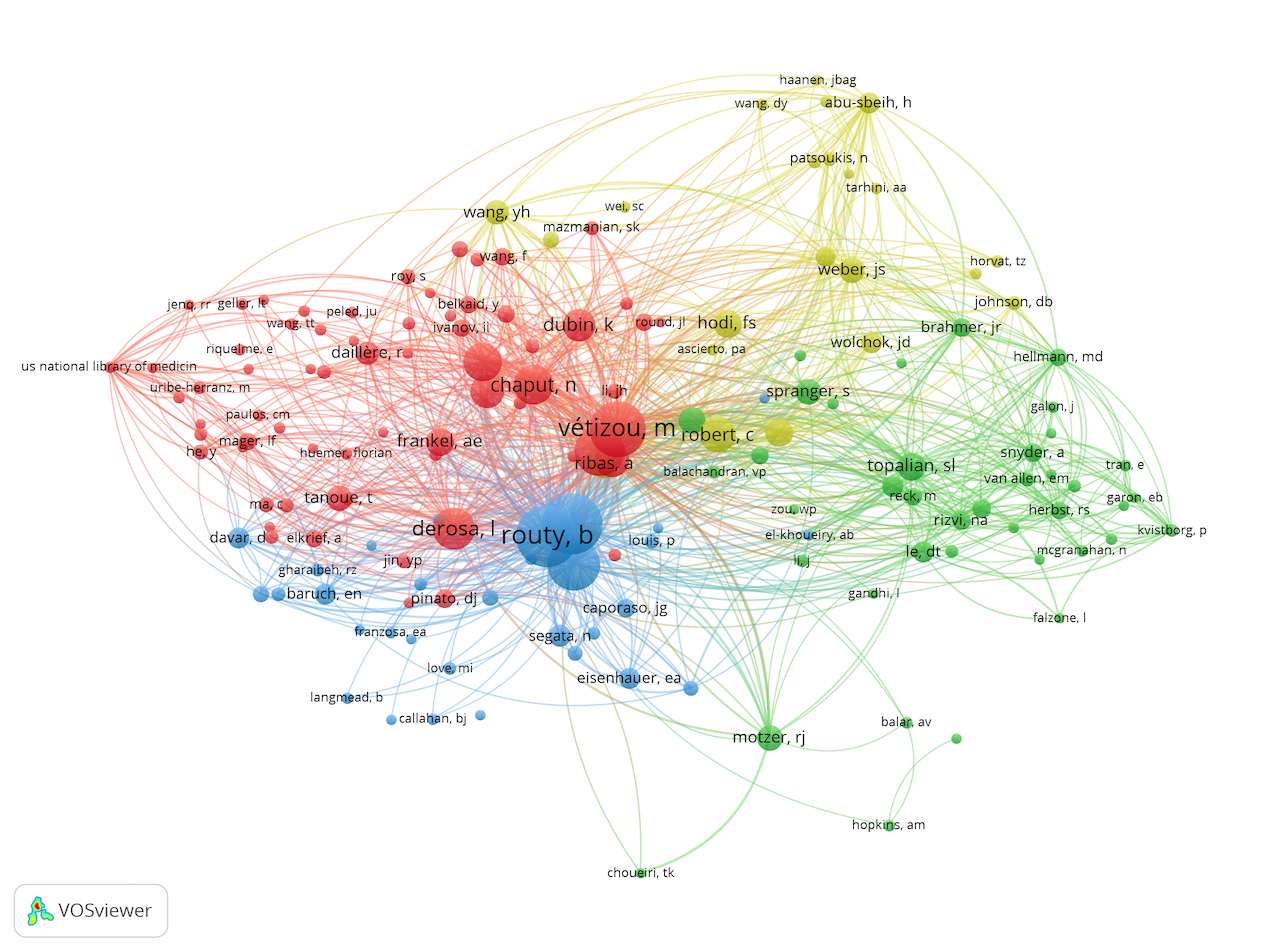

Supplement: Supplementary file 1 [file DataSheet1.zip › Supplementary Material Presentation/Figure5C.tiff]

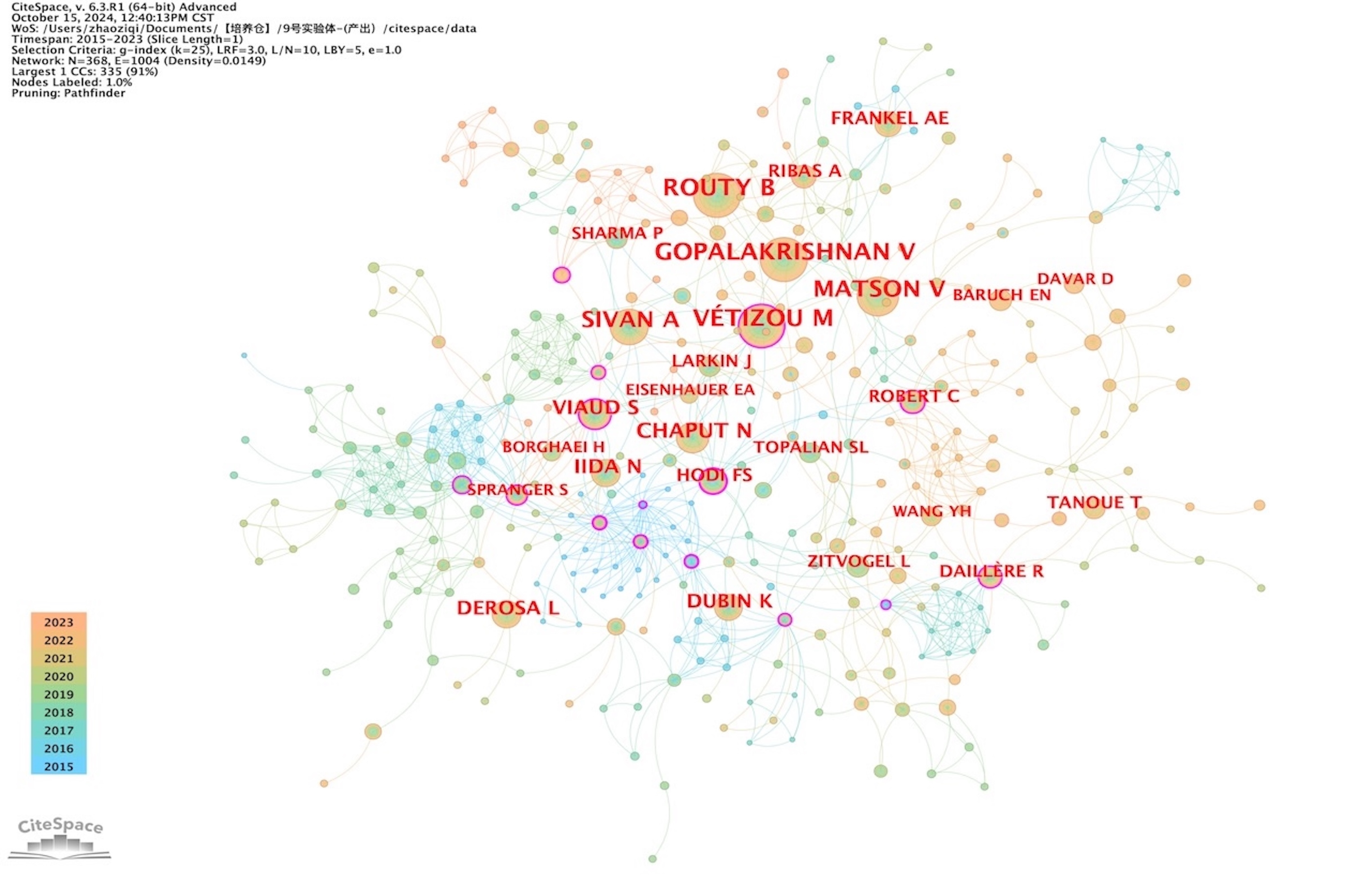

Supplement: Supplementary file 1 [file DataSheet1.zip › Supplementary Material Presentation/Figure5D.jpeg]

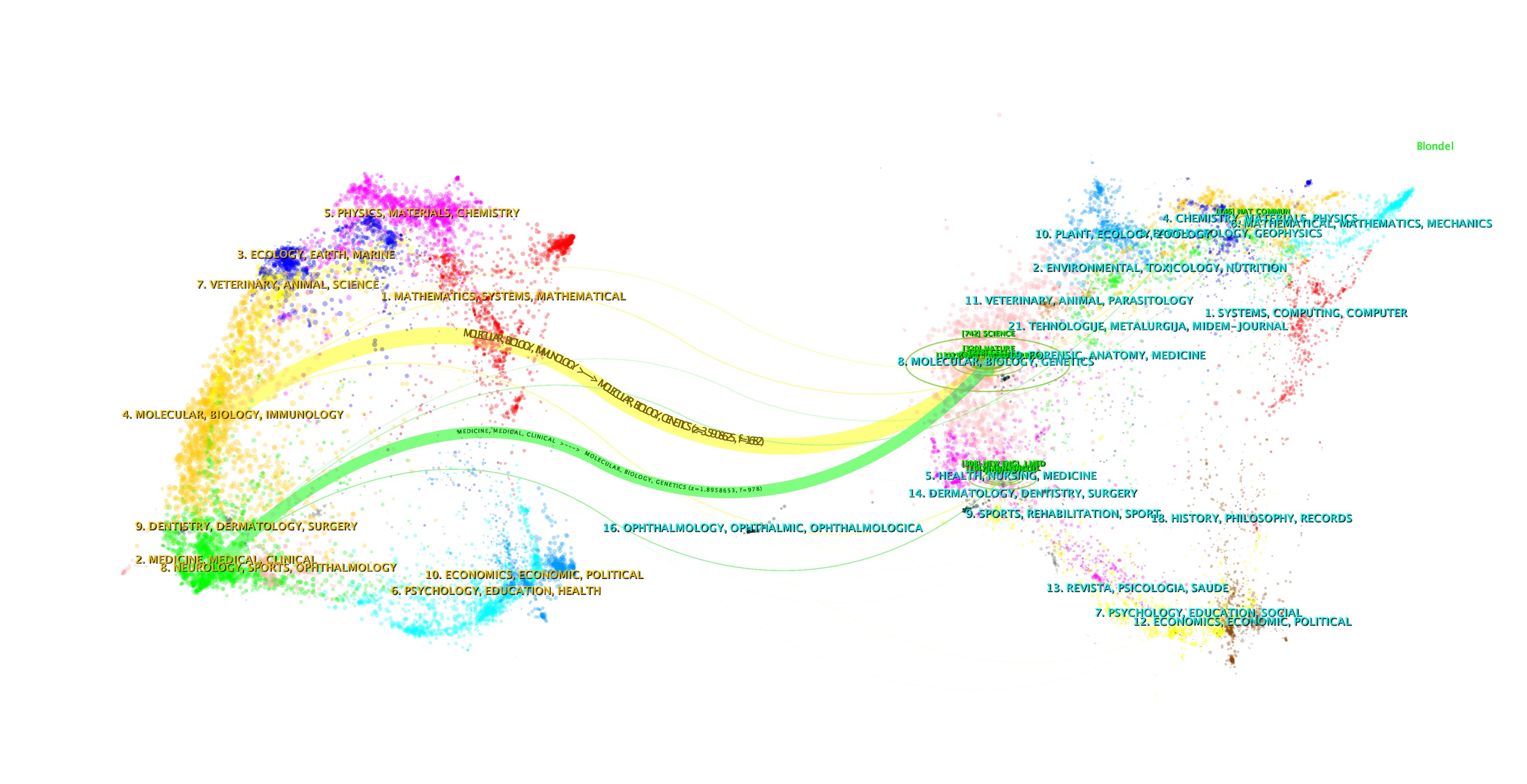

Supplement: Supplementary file 1 [file DataSheet1.zip › Supplementary Material Presentation/Figure6.jpeg]

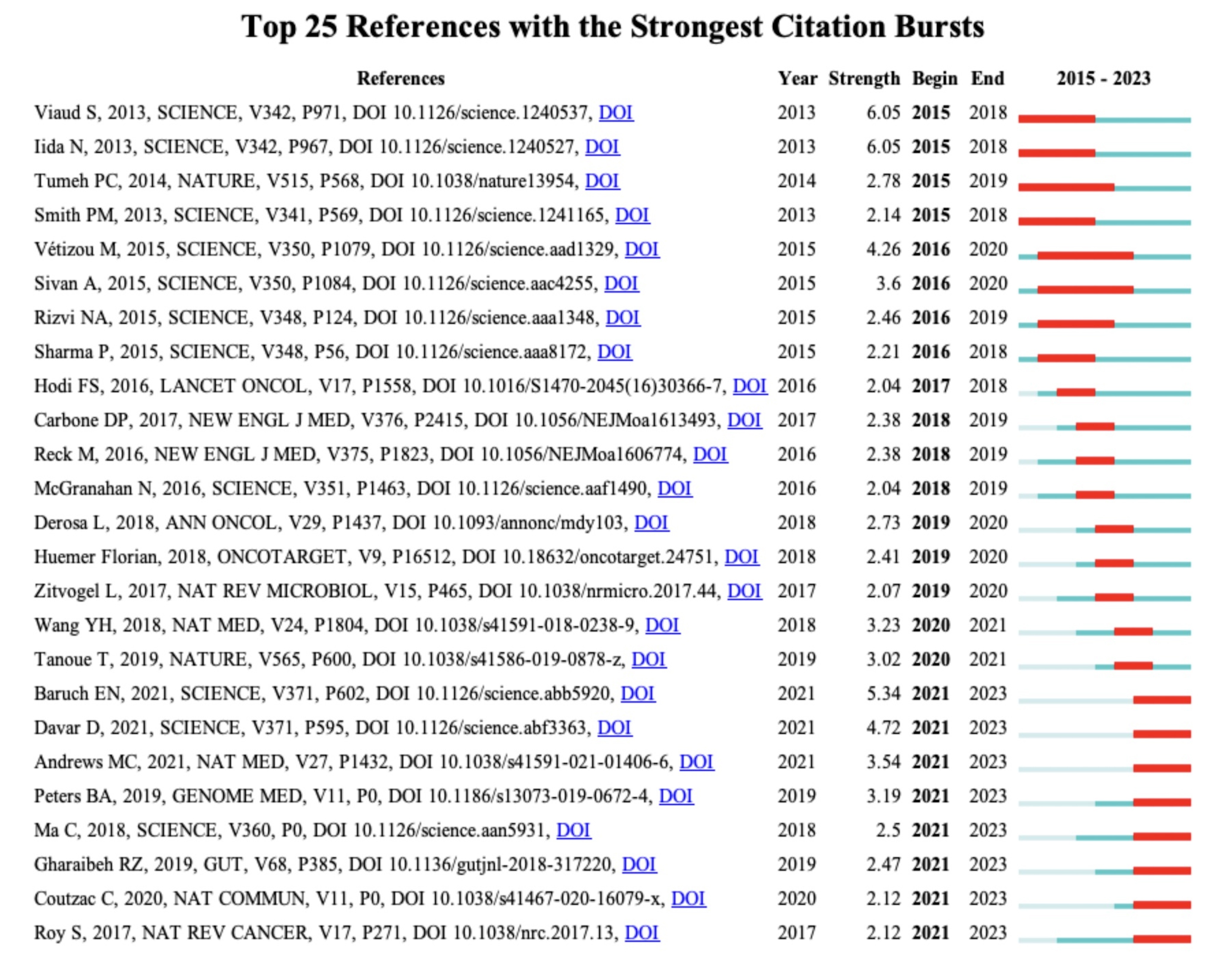

Supplement: Supplementary file 1 [file DataSheet1.zip › Supplementary Material Presentation/Figure7A.jpeg]

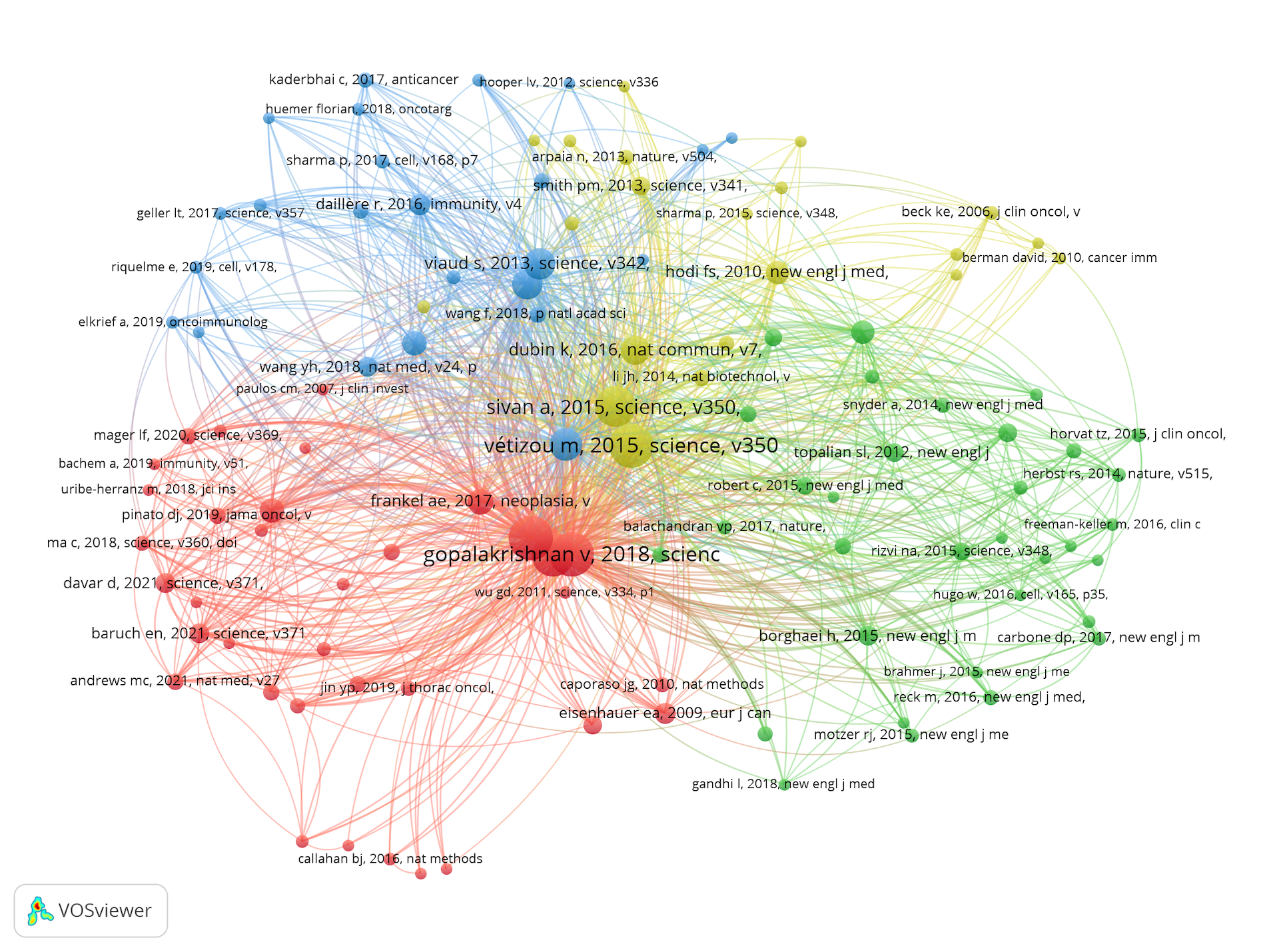

Supplement: Supplementary file 1 [file DataSheet1.zip › Supplementary Material Presentation/Figure7B.tiff]

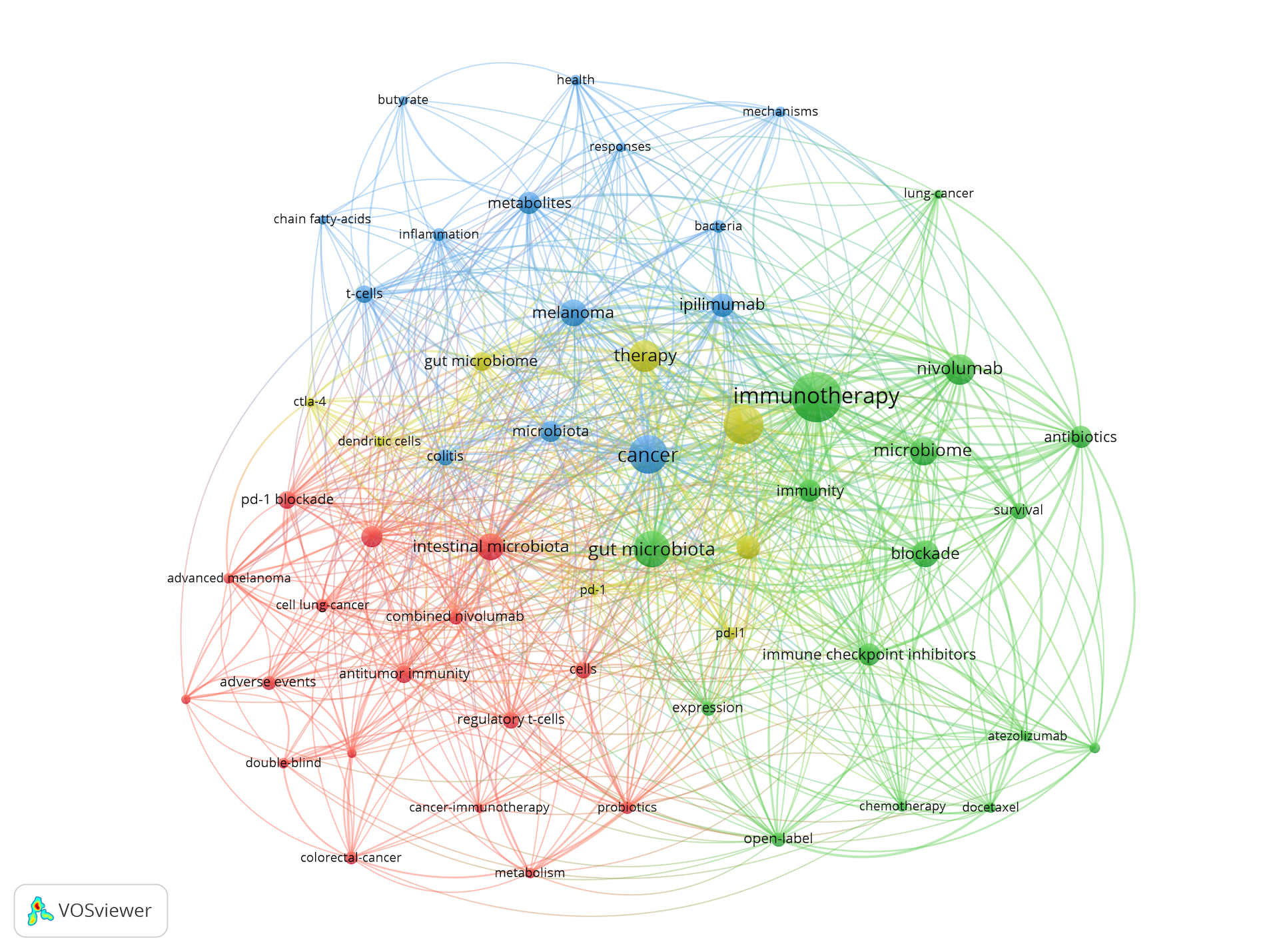

Supplement: Supplementary file 1 [file DataSheet1.zip › Supplementary Material Presentation/Figure8A.tiff]

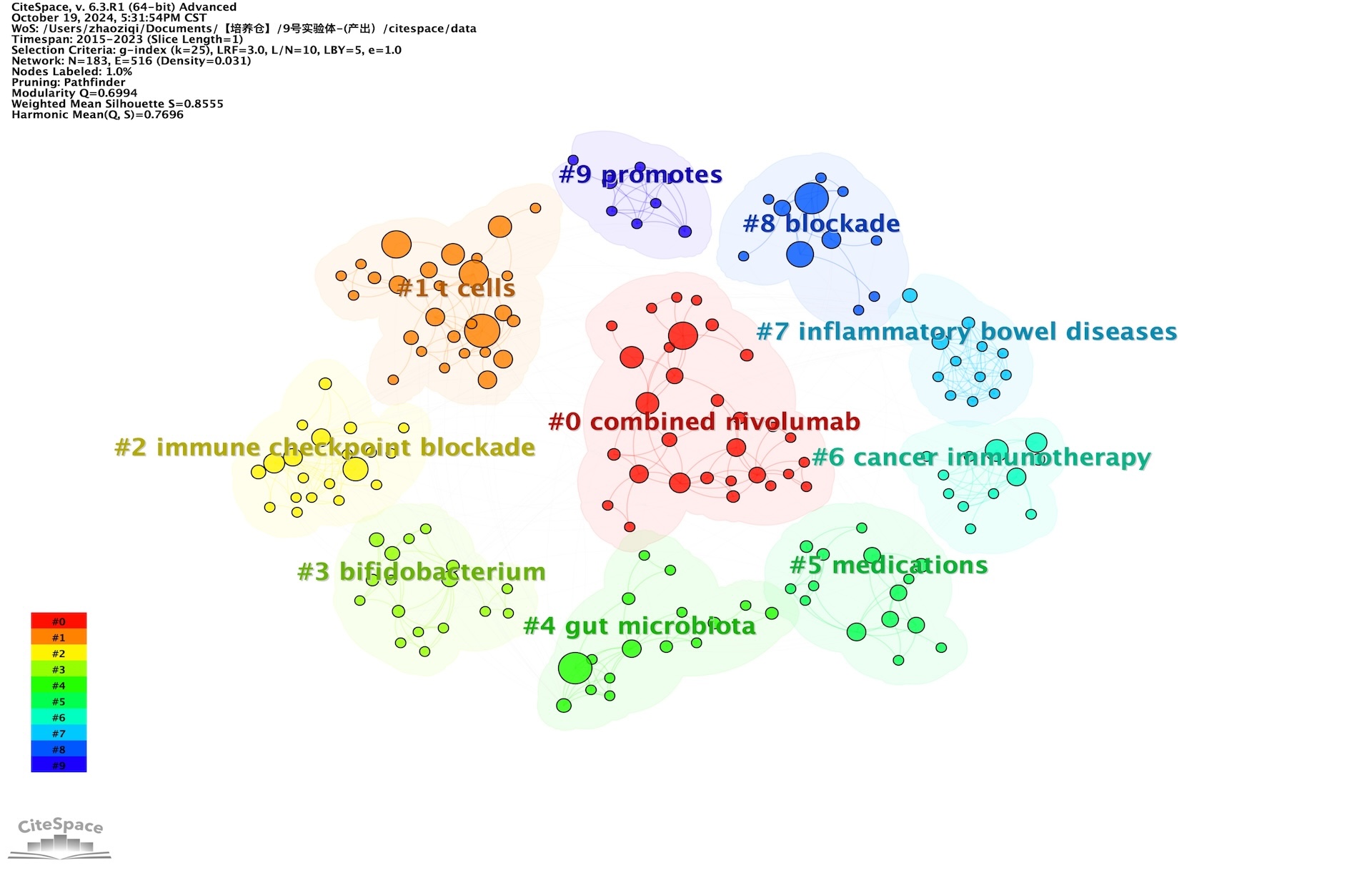

Supplement: Supplementary file 1 [file DataSheet1.zip › Supplementary Material Presentation/Figure8B.jpeg]

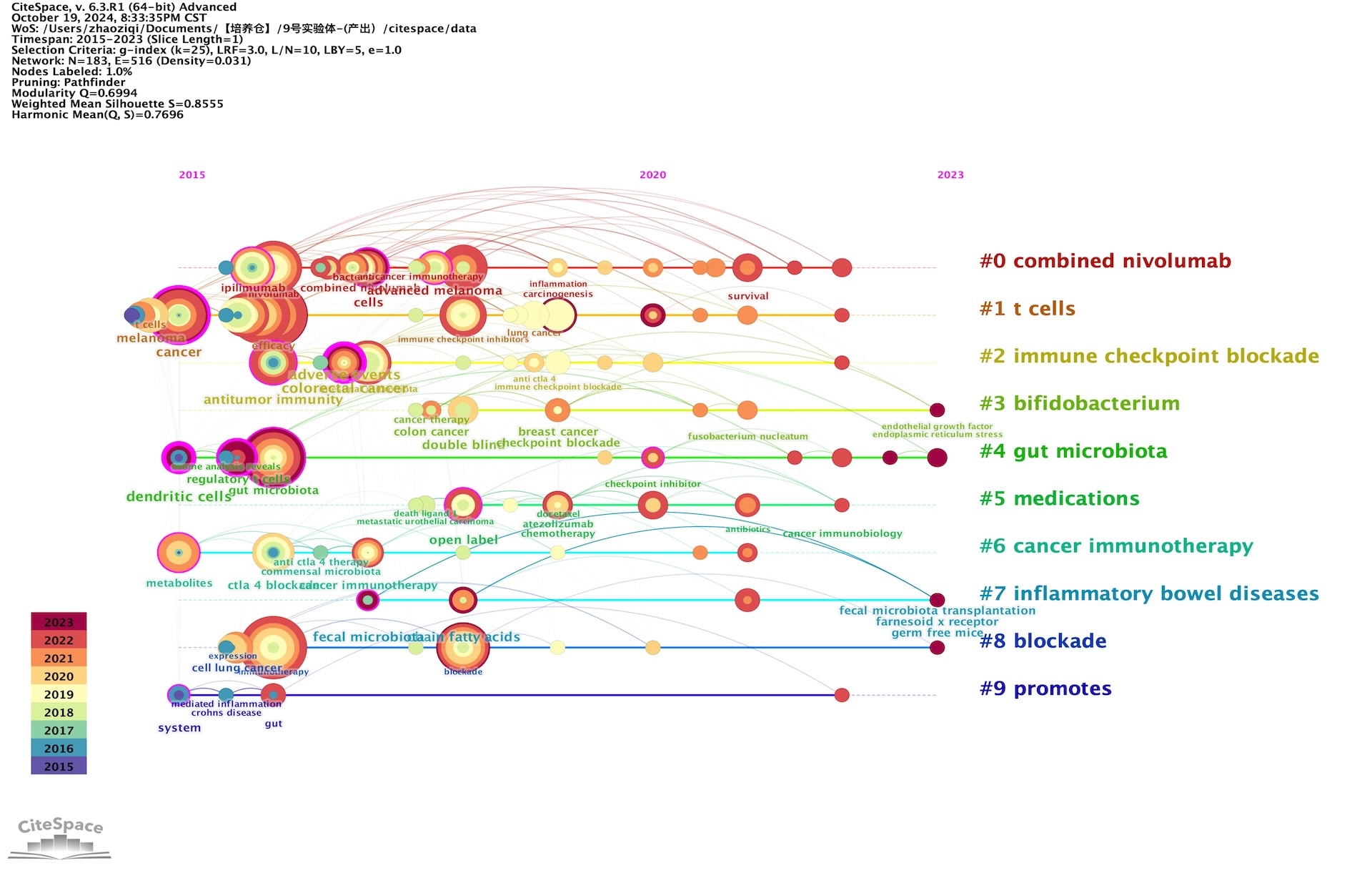

Supplement: Supplementary file 1 [file DataSheet1.zip › Supplementary Material Presentation/Figure8C.jpeg]

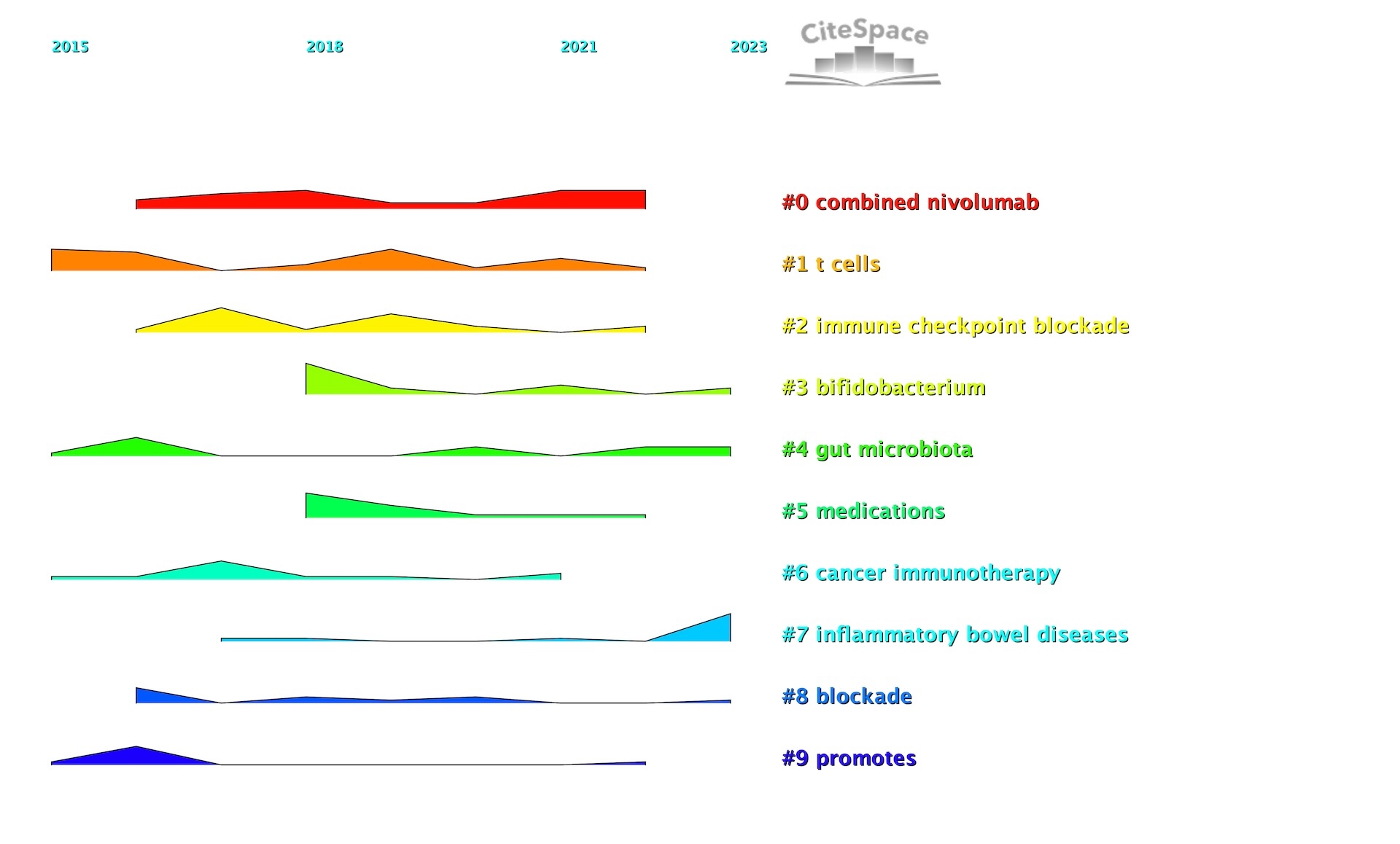

Supplement: Supplementary file 1 [file DataSheet1.zip › Supplementary Material Presentation/Figure8D.jpeg]

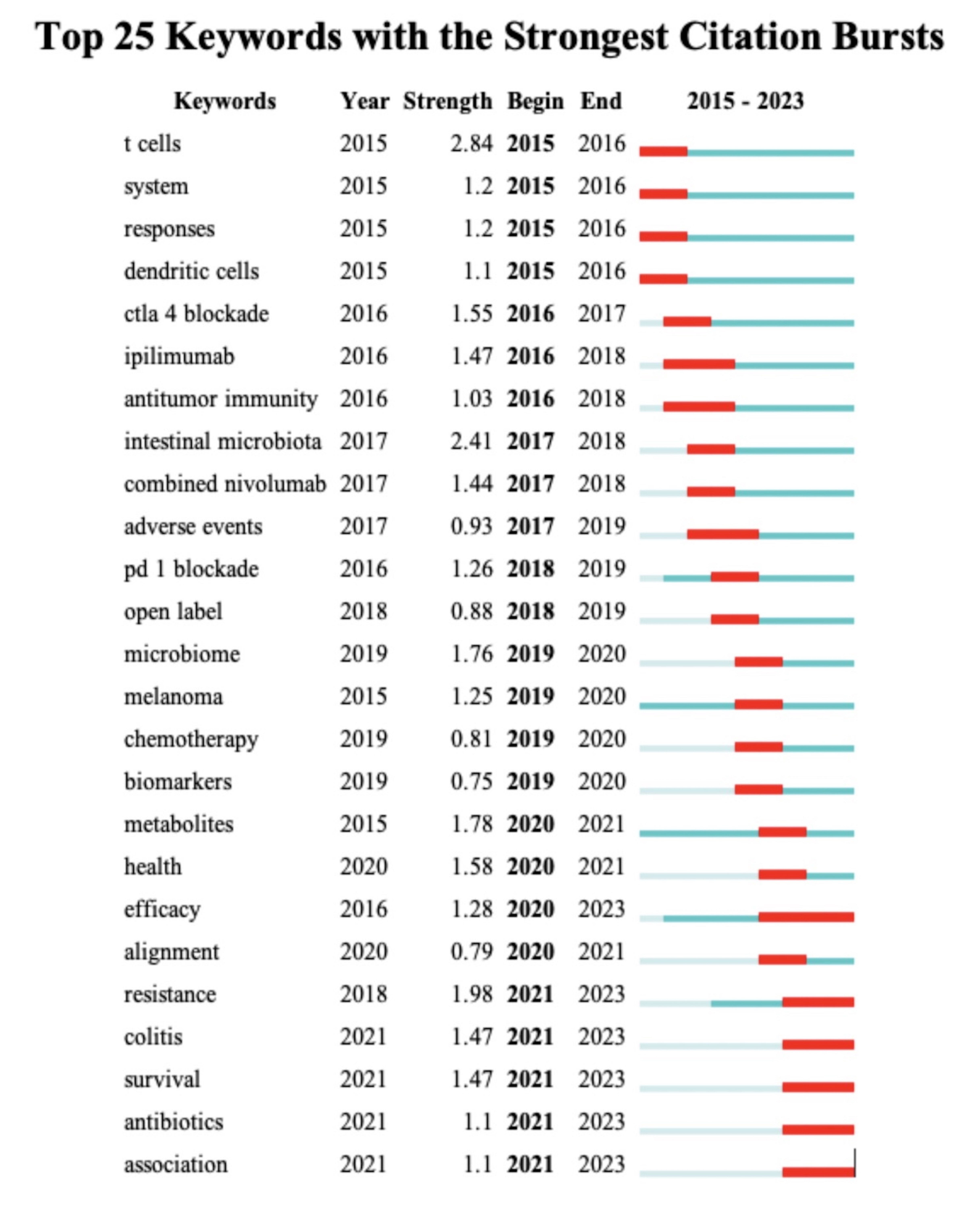

Supplement: Supplementary file 1 [file DataSheet1.zip › Supplementary Material Presentation/Figure9.jpeg]
